# Prioritising surveillance for alien organisms transported as stowaways on ships travelling to South Africa

**DOI:** 10.1371/journal.pone.0173340

**Published:** 2017-04-05

**Authors:** Katelyn T. Faulkner, Mark P. Robertson, Mathieu Rouget, John R. U. Wilson

**Affiliations:** 1 Invasive Species Programme, South African National Biodiversity Institute, Kirstenbosch Research Centre, Claremont, South Africa; 2 Centre for Invasion Biology, Department of Zoology and Entomology, University of Pretoria, Hatfield, South Africa; 3 CIRAD, UMR PVBMT, La Réunion, France; 4 Centre for Invasion Biology, Department of Botany and Zoology, Stellenbosch University, Matieland, South Africa; Maurice Lamontagne Institute, CANADA

## Abstract

The global shipping network facilitates the transportation and introduction of marine and terrestrial organisms to regions where they are not native, and some of these organisms become invasive. South Africa was used as a case study to evaluate the potential for shipping to contribute to the introduction and establishment of marine and terrestrial alien species (i.e. establishment debt) and to assess how this varies across shipping routes and seasons. As a proxy for the number of species introduced (i.e. ‘colonisation pressure’) shipping movement data were used to determine, for each season, the number of ships that visited South African ports from foreign ports and the number of days travelled between ports. Seasonal marine and terrestrial environmental similarity between South African and foreign ports was then used to estimate the likelihood that introduced species would establish. These data were used to determine the seasonal relative contribution of shipping routes to South Africa’s marine and terrestrial establishment debt. Additionally, distribution data were used to identify marine and terrestrial species that are known to be invasive elsewhere and which might be introduced to each South African port through shipping routes that have a high relative contribution to establishment debt. Shipping routes from Asian ports, especially Singapore, have a particularly high relative contribution to South Africa’s establishment debt, while among South African ports, Durban has the highest risk of being invaded. There was seasonal variation in the shipping routes that have a high relative contribution to the establishment debt of the South African ports. The presented method provides a simple way to prioritise surveillance effort and our results indicate that, for South Africa, port-specific prevention strategies should be developed, a large portion of the available resources should be allocated to Durban, and seasonal variations and their consequences for prevention strategies should be explored further.

## Introduction

Global shipping facilitates the transportation and introduction of organisms to regions where they are not native, and some of these organisms become invasive. Through the release of ballast water [1,2] and biofouling [3], shipping has become the dominant vector for the global spread of alien marine organisms [4–6]. Terrestrial organisms are also often transported and introduced through shipping [4,7,8]. For example, insects and molluscs are transported in shipping containers [7–9], and the recent spread of the mosquito and disease vector, *Aedes albopictus*, has largely been attributed to the shipping of tyres around the world (water held in tyres provide refuges for larvae) [10]. Although other transportation networks facilitate the spread of alien organisms (e.g. the global airline network), the structure of the global shipping network (highly clustered and densely connected, with small ports connected only to larger ports) might make this transportation network particularly prone to spreading alien species [11]. In line with this, in the USA more insects (individuals and species) have been intercepted at maritime ports of entry than at airports and land border crossings [9].

In some regions, efforts have been made to reduce introductions facilitated by shipping (e.g. in Australia the discharge of unmanaged ballast water is prohibited [12], the vessel or goods it transports can be inspected for pests by biosecurity officers [13], and, although not currently addressed in Australian legislation, ship operators are encouraged to follow National Biofouling Management Guidelines [e.g. 14]). Where these efforts have been implemented and enforced the risk of invasion has often been reduced [15]. However in many parts of the world, the number of ship-facilitated introductions continues to increase (e.g. marine species in Europe [16] and mosquitos in New Zealand [17]). Although these increases might be due to increased shipping intensities, they could also be sampling artefacts, due to either an increase in the number and intensity of surveys for alien species, or the time lag between the introduction of alien species and their detection (i.e. invasion debt) [6,18–21].

Although shipping intensity and the amount of cargo transported by ships continues to increase, the resources available to develop and implement prevention strategies are limited [7,9,22]. For improved biosecurity outcomes (invasion policy and management), prevention strategies must prioritise introduction pathways, species, and sites [23–26]. Such prioritised prevention strategies would not only be more effective [23,27,28], but management costs might be lower if only high risk ports of entry or shipping routes are identified and appropriately targeted than if uniform strategies are employed [29].

Marine and terrestrial introductions facilitated by shipping appear to vary geographically, with some regions donating and receiving more organisms than others [7,17,30]. Theoretical studies on shipping-facilitated marine invasions have highlighted that, due to high propagule pressure (i.e. the number of individuals introduced and/or the number of introduction events for any particular species [31]) and colonisation pressure (i.e. the number of species introduced [32]), busy ports are likely to be more prone to invasions [11,33], and routes with high shipping traffic are more likely to result in introductions and invasions than those with lower traffic [21,34,35]. Consistent with these marine predictions, a large number of terrestrial organisms have been intercepted at the busiest ports of New Zealand and the USA [7,17]. Additionally, shipping routes that connect ports that are geographically close and environmentally similar are more likely to introduce future invaders, as organisms are likely to survive transportation (due to short voyage duration [2,6,36,37]) and establish in the recipient region [35,38,39]. Ports where many species are introduced (i.e. high colonisation pressure) and where the likelihood of establishment is high will have a high establishment debt (i.e. a large number of species will establish [40]). Finally, as the amount of shipping traffic and the environmental similarity between ports varies seasonally, so too will the estimated number of species that will be introduced and establish [7,33,35,39]. The contribution of particular shipping routes to establishment debt (and so a country’s invasion debt) will therefore vary, but if we can predict these variations, it might be possible to improve prevention strategies.

Studies that have attempted to predict shipping-facilitated invasions have largely focused on marine invasions (but work has been done for some terrestrial species, like mosquitos [10] and the gypsy moth [39]) and are often global in extent [11,34,35,41]. Although some of these studies have assessed the risk of shipping-facilitated invasions in specific regions, these have largely focused on developed regions that are already highly invaded (e.g. the Great Lakes [38,42] and the Baltic Sea [43]; but cf. predictions for Indonesia [44,45]). Here we aimed to evaluate the contribution of shipping-facilitated stowaway invasions to a developing nation’s marine and terrestrial establishment debt, where stowaways are defined as organisms introduced on or in a transport vector [4]. South Africa was used as a case study as there has been a recent shift in its trade partners [46], and thus this country might be particularly at risk of new introductions and invasions [47–49]. In an effort to identify priorities for prevention strategies we specifically assessed the relative contribution of shipping routes to the marine and terrestrial establishment debt of South Africa, and undertook a preliminary exploration of whether this contribution varies seasonally. Furthermore, we identified species that are known to be invasive elsewhere and that might be introduced to South African ports following transportation along high risk shipping routes (routes with a high relative contribution to South Africa’s establishment debt).

## Materials and methods

### Contribution of shipping to South Africa’s establishment debt

We assumed that the routes that contribute the most to establishment debt are those that: a) have high shipping traffic (measured as the number of ship visits); b) have short voyage duration (measured as the number of days travelled by a ship); and c) link environmentally similar ports (evaluated using environmental matching). These three factors were combined to determine the relative contribution of shipping routes to establishment debt and preliminarily evaluate whether this contribution varies seasonally (see below). Multiple sources of variation may influence the validity of the aforementioned assumptions and our assumptions might not hold in all instances. For example, although the survival of organisms in ballast tanks is inversely related to voyage duration [36,37,50], this relationship might not hold for organisms transported in resting stages [51,52]. Furthermore, ship types will differ with regards to ballast water discharge patterns and transported merchandise, which will in turn cause variations in the introduction of marine and terrestrial species [10,50]. It is, however, important to note that this analysis considered many important sources of variation (e.g. duration of voyage and source region) and that it has been shown that where detailed propagule pressure data are lacking (e.g. ballast water, biofouling and cargo offload data), ship traffic data are a useful proxy [10,33,45].

#### Number of ship visits and days travelled

The International Maritime Organisation requires all ships to carry an Automatic Identification System. The Automatic Identification System records information on the identity and location of ships travelling around the globe. Access to these data were obtained through the Sea-Web Movements database provided by IHS Inc. (http://www.sea-web.com/seaweb_movements_module.html). The database was used to obtain information for all vessels that visited five large South African ports (Richards Bay, Durban, Port Elizabeth, Cape Town and Saldanha Bay; see S1 Fig) during 2014. South Africa is a particularly good place to study variation in the risks posed by stowaways as the five selected ports not only have the highest number of visiting vessels (S2 Fig) but also have different marine environments and climates (S1 Fig). Vessels that were unlikely to have undertaken international journeys were excluded from the dataset (459 unique vessels, including tugs, fishing vessels and search and rescue vessels). For all other vessels (3935 unique vessels), data on all ship movements made between 1 October 2013 and 31 December 2014 were extracted from the database. These data included information on all the ports visited in the previous 90 days (see S1 Table for information on the foreign ports included in the analysis) as well as the arrival and departure dates for each port visit (314 472 movement records). For some records vital data were missing (e.g. the name of the port and/or country visited). If these data could not be determined (e.g. by evaluating the ships’ movements before and after the record in question) the record was excluded from the dataset (42 763 movement records (i.e. 13.6%)). The extracted ship movement data were then used to construct the trajectory of each ship (i.e. the ports visited sorted by date and time of arrival). For each vessel, all visits to the five South African ports in 2014 and all the ports visited (South African or foreign) during the previous 90 days were identified. Thus it was assumed that an organism could be introduced from any of the ports that a vessel had visited during the 90 days prior to arriving at a South African port. It was also assumed that organisms are unlikely to be introduced alive if transported for more than 90 days. As an example, the propagule pressure of organisms transported by ballast water is estimated to decline by 80–99% in 25 days, but estimates vary across organisms and vectors [2]. As such our 90 days cut-off represents a cautious estimate. In some cases, vessels completed a round trip within 90 days (i.e. a ship left South Africa but returned within 90 days). In these instances we made the simplifying assumption that all transported organisms would have been introduced during the previous visit to South Africa and only ship movements that occurred after this preceding visit were included. These ship movement data were used to calculate (1) the number of ships that visited each South African port from each foreign port, and (2) the average number of days a ship travelled between each foreign port and each South African port.

Seasonal results were obtained by summing the number of ships that visited a South African port from a foreign port during months that fall into southern hemisphere seasons (i.e. June–August is winter; September–November is spring; December–February is summer; March–May is autumn). Similarly, the average number of days travelled between ports for months that fall into southern hemisphere seasons were calculated.

#### Environmental matching

The climatic distances between ports were calculated using gridded monthly mean temperature and precipitation data from the CRU CL 2.0 10 minute dataset [53]. Similarly, the marine environmental distances between ports (marine ports only, inland ports were excluded) were calculated using gridded monthly mean sea surface temperature and monthly mean salinity data [as recommended in 29] from the 2013 World Ocean Atlas 15 minute dataset [54,55]. As large biases in satellite-derived sea surface temperature data have been reported [56], the World Ocean Atlas database was specifically used as it provides *in situ* marine environmental information [54]. In both cases, for each port the environmental data from the relevant cell were extracted in ArcMap 10 (ESRI, Red-lands California). In their evaluation of environmental distance metrics for alien species risk assessments, Bradie et al. [57] demonstrated that unweighted Euclidean distance performed the best of the metrics, thus this metric was used to determine the climatic and marine environmental distances between ports. To ensure that climate (temperature and precipitation) and marine environment (sea surface temperature and salinity) variables were equally weighted when calculating Euclidean distance we followed standard protocol [29] and the extracted data for each variable were transformed in R version 3.0.2 [58] such that the data had a mean of 0 and a standard deviation of 1. For each month, the Euclidean distance in a two dimensional climate space (mean temperature and precipitation) between each port was calculated using the ‘stats’ package in R [58] and a distance matrix was obtained (see Figs A–C in S3 Fig for examples of climate pairwise-comparison results). Following the same methods, the Euclidean distance in a two dimensional marine environment space (mean sea surface temperature and salinity) between each port was calculated for each month (see Figs A–C in S4 Fig for examples of marine environment pairwise-comparison results). Only the pairwise comparisons between the five selected South African ports and foreign ports were retained and used in the analysis (i.e. Euclidean distance results for pairs of ports not in South Africa were removed).

Seasonal results were obtained by calculating the average environmental distance (climate and marine environment) between ports for months that fall into southern hemisphere seasons (see above section for definitions).

#### Relative contribution of shipping routes to establishment debt and shipping route ranking

As variation in the contribution of shipping routes to establishment debt in terms of both relative contribution and ranking should be considered when identifying priorities for prevention strategies, variation in both factors were taken into account in the analysis.

Let *s* ∈ *S*, where *S* is a set of seasons and let *r* ∈ *R*, where *R* is a set of shipping routes. Let *v*_*r*,*s*_ ∈ *V*, where *V* is a *r* × *s* matrix, let *d*_*r*,*s*_ ∈ *D*, where *D* is a *r* × *s* matrix and let *e*_*r*,*s*_ ∈ *E*, where *E* is a *r* × *s* matrix (see Table 1). The seasonal number of ship visits for each route from a foreign port to a South African port *v*_*r*,*s*_ were summed to obtain the total number of ship visits for each route *V*_*r*_ (formula 1, Table 1). Similarly, the seasonal number of days travelled *d*_*r*,*s*_ and environmental distance *e*_*r*,*s*_ between each foreign port and each South African port were averaged to obtain the average number of days travelled *D*_*r*_ and average environmental distance *E*_*r*_ for each shipping route connecting a foreign port to a South African port (formulae 2 and 3, Table 1). As the relative importance of the number of ship visits, the number of days travelled and environmental distance in determining invasions is not fully understood, these metrics were assigned equal weight when calculating the relative contribution of the shipping routes to establishment debt [e.g. 10]. To achieve this, the min-max method [59] was used to rescale each variable such that the values ranged between 0 and 1. For example, rescaled ship visit values for each route ${\hat{V}}_{r}$ were the original values *V*_*r*_ minus the minimum value *min*(*V*_*r*_), divided by the difference between the maximum value *max*(*V*_*r*_) and the minimum value *min*(*V*_*r*_) (formula 4, Table 1). The days travelled *D*_*r*_ and environmental distance *E*_*r*_ data were similarly rescaled (formulae 5 and 6, Table 1). For each route, the complement of the rescaled number of days travelled ${\bar{D}}_{r}$ and environmental distance ${\bar{E}}_{r}$ values were calculated such that high values became low and vice versa (formulae 7 and 8, Table 1). For example, an environmental distance of one was converted to an environmental similarity of zero. The relative contribution of each shipping route to establishment debt *I*_*r*_ was determined by taking the product these values and the rescaled number of ship visits (formula 9, Table 1). For each South African port, the shipping routes from foreign ports were then ranked based on their relative contribution to establishment debt (referred to as ‘route ranking’).

**Table 1:** The components considered when calculating the relative contribution of the shipping routes to establishment debt, the notation used to refer to each component and, where applicable, the formulae used to calculate the components.

Notation	Component	Formula	
*v*_*r*,*s*_	Seasonal number of ship visits for each route		
*d*_*r*,*s*_	Seasonal number of days travelled for each route		
*e*_*r*,*s*_	Seasonal environmental distance for each route		
*n*	Total number of seasons in set *S*		
*V*_*r*_	Number of ship visits for each route	$V_{r}=\sum_{s\in S}v_{r,s}$	(1)
*D*_*r*_	Average number of days travelled for each route	$D_{r}=\frac{\sum_{s\in S}d_{r,s}}{n}$	(2)
*E*_*r*_	Average environmental distance for each route	$E_{r}=\frac{\sum_{s\in S}e_{r,s}}{n}$	(3)
${\hat{V}}_{r}$	Rescaled number of ship visits for each route	${\hat{V}}_{r}=(V_{r}-min(V_{r}))/(max(V_{r})-min(V_{r}))$	(4)
${\hat{D}}_{r}$	Rescaled average number of days travelled for each route	${\hat{D}}_{r}=(D_{r}-min(D_{r}))/(max(D_{r})-min(D_{r}))$	(5)
${\hat{E}}_{r}$	Rescaled average environmental distance for each route	${\hat{E}}_{r}=(E_{r}-min(E_{r}))/(max(E_{r})-min(E_{r}))$	(6)
${\bar{D}}_{r}$	Complement of rescaled number of days travelled for each route	${\bar{D}}_{r}=1-{\hat{D}}_{r}$	(7)
${\bar{E}}_{r}$	Complement of rescaled environmental distance for each route	${\bar{E}}_{r}=1-{\hat{E}}_{r}$	(8)
*I*_*r*_	Relative contribution of each route to establishment debt	$I_{r}={\hat{V}}_{r}\times {\bar{D}}_{r}{\times \bar{E}}_{r}$	(9)
$v_{p,s}^{*}$	Seasonal number of ship visits for each route related to a specific South African port		
$d_{p,s}^{*}$	Seasonal number of days travelled for each route related to a specific South African port		
$e_{p,s}^{*}$	Seasonal environmental distance for each route related to a specific South African port		
${\hat{v}}_{p,s}^{*}$	Rescaled seasonal number of ship visits for each route related to a specific South African port	${\hat{v}}_{p,s}^{*}=(v_{p,s}^{*}-min(V^{*}))/(max(V^{*})-min(V^{*}))$	(10)
${\hat{d}}_{p,s}^{*}$	Rescaled seasonal number of days travelled for each route related to a specific South African port	${\hat{d}}_{p,s}^{*}=(d_{p,s}^{*}-min(D^{*}))/(max(D^{*})-min(D^{*}))$	(11)
${\hat{e}}_{p,s}^{*}$	Rescaled seasonal environmental distance for each route related to a specific South African port	${\hat{e}}_{p,s}^{*}=(e_{p,s}^{*}-min(E^{*}))/(max(E^{*})-min(E^{*}))$	(12)
${\bar{d}}_{p,s}^{*}$	Complement of rescaled seasonal number of days travelled for each route related to a specific South African port	${\bar{d}}_{p,s}^{*}=1-{\hat{d}}_{p,s}^{*}$	(13)
${\bar{e}}_{p,s}^{*}$	Complement of rescaled seasonal environmental distance for each route related to a specific South African port	${\bar{e}}_{p,s}^{*}=1-{\hat{e}}_{p,s}^{*}$	(14)
$I_{p,s}^{*}$	Seasonal relative contribution of each route to the establishment debt of a specific South African port	$I_{p,s}^{*}={\hat{v}}_{p,s}^{*}\times {\bar{d}}_{p,s}^{*}\times {\bar{e}}_{p,s}^{*}$	(15)

Let *p* ∈ *P* where *P* is the set of routes to a specific South African port. We define V* ⊂ *V* where V* is a *p* × *s* matrix, with $v_{p,s}^{*}\in V^{*}$. Similarly let D* ⊂ *D* where D* is a *p* × *s* matrix, with $d_{p,s}^{*}\in D^{*}$, and let E* ⊂ *E* where E* is a *p* × *s* matrix, with $e_{p,s}^{*}\in E^{*}$. Following the methods discussed above, for each South African port, the seasonal number of ship visits $v_{p,s}^{*}$, number of days travelled $d_{p,s}^{*}$ and environmental distance results $e_{p,s}^{*}$ for each shipping route from a foreign port were rescaled and used to calculate the relative seasonal contribution of routes from foreign ports to establishment debt $I_{p,s}^{*}$ (see formulae 10–15, Table 1). For each South African port and season, these shipping routes were then ranked based on their relative contribution to establishment debt (referred to as ‘route ranking’).

#### Analysis

Variation among the South African ports was evaluated using a Kruskal-Wallis test with port as the independent variable, and the relative contribution of shipping routes to establishment debt as the dependent variable. Multiple comparisons among ports were done using pairwise Mann-Whitney U tests with the Holm correction [60, 61]. These non-parametric methods are appropriate in instances such as this where the data are not normally distributed and are difficult to transform [60].

The similarity of the route rankings for the South African ports was evaluated using Kendall’s coefficient of concordance (Kendall’s W, 'vegan' package in R [62]). As our data were right skewed with many zeros, this test was selected as it has previously been used to analyse species abundance data which are similarly distributed [63]. Following Legendre [63] the Hellinger transformation was used and an overall test of independence was performed on the transformed data. The null hypothesis of Kendall’s coefficient of concordance is that the rankings are independent or random and thus if the results for the overall test are significant, then the route rankings for the South African ports are concordant with one another [63,64]. Following the methods discussed above, for each South African port a Kruskal-Wallis test and Kendall’s coefficient of concordance were used to evaluate seasonal variation in the relative contribution of the shipping routes to establishment debt and route rankings. Results were plotted and mapped in R version 3.0.2 [58] using the packages ‘maptools’ [65], ‘RColorBrewer’ [66] and ‘geosphere’ [67].

### Comparison with field data

Unfortunately it was not possible to use interception data to validate our results, as such data are not kept by inspectors at South Africa’s ports of entry. However, similar studies of shipping-facilitated invasions have validated their results using field data. For example in their global study, Seebens et al. [35] showed that for various ecoregions the expected number of invasions agreed with the number of invasion events. Similarly, we compared our results for the marine environment to data from South African catalogues [i.e. 68,69] on marine alien species that have been recorded in South Africa. As Mead et al. [69] does not provide species lists for each South African port but rather for each biogeographical region, we evaluated the number of alien species that occur in the biogeographical region associated with each port. Please note that for this analysis the biogeographical regions as classified by Mead et al. [69] were used (S5 Fig) and that these regions are not the same as the bioregions of Sink et al. [70] (S1 Fig). Furthermore, the South African catalogue data [68] were used to determine the number of marine species introduced to South Africa from different regions and to evaluate whether these patterns have changed over time. Unfortunately, as port-level field data are not available for terrestrial species, this analysis could not be undertaken for the terrestrial component of the study.

### Watch list species associated with high risk shipping routes

There are 35 marine and 365 terrestrial invasive species on the watch list developed for South Africa by Faulkner et al. [71]. These species have not yet been introduced to South Africa, but have a history of invasion and occur in environmentally similar regions with trade and tourism links to South Africa. As such, these species might be introduced and become invasive in South Africa in the future. For each South African port, we aimed to identify marine and terrestrial watch list species that might be transported and introduced by ships travelling along high risk routes (i.e. routes that have a high relative contribution to establishment debt). To achieve this, for each South African port we identified the twenty shipping routes from foreign ports (referred to as ‘source ports’) that have the highest relative contribution to marine and terrestrial establishment debt. Distribution data from the native and introduced range of each watch list species were obtained from the Global Biodiversity Information Facility. These distribution data were used to identify the terrestrial and marine watch list species that occur in the Köppen-Geiger climate zones [72] or Bailey ecoregion divisions [73] that are associated with the source ports of the high risk routes. Thus it was assumed that if a species occurs in the climate zone or marine ecoregion in which the source port of a high risk route is found then the species has a good chance of being transported from the source port to South Africa. Not all of the watch list species are introduced through shipping. Thus we obtained pathway of introduction information from the Global Invasive Species Database for the species that are associated with the source ports of the high risk routes and included in the analysis only those species that have a history of shipping-facilitated introduction. For each South African port, a list of the watch list species that might be transported to the port along each high risk route was obtained and results were mapped in R version 3.0.2 [58].

## Results

### Contribution of shipping to South Africa’s establishment debt

**Fig 1:**
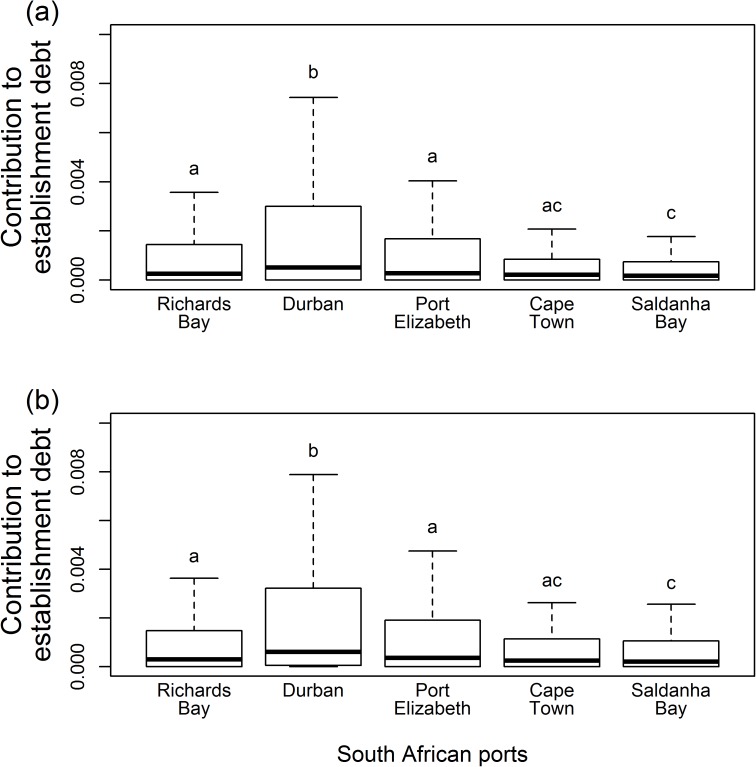
The relative contribution of shipping routes from foreign ports to South African ports to (a) marine (Kruskal-Wallis: *χ*^*2*^ = 59.33, df = 4, *P* < 0.001) and (b) terrestrial (Kruskal-Wallis: *χ*^*2*^ = 83.23, df = 4, *P* < 0.001) establishment debt. Different lower case letters indicate significant differences amongst the South African ports. Boxplots represent median and interquartile range.

**Fig 2:**
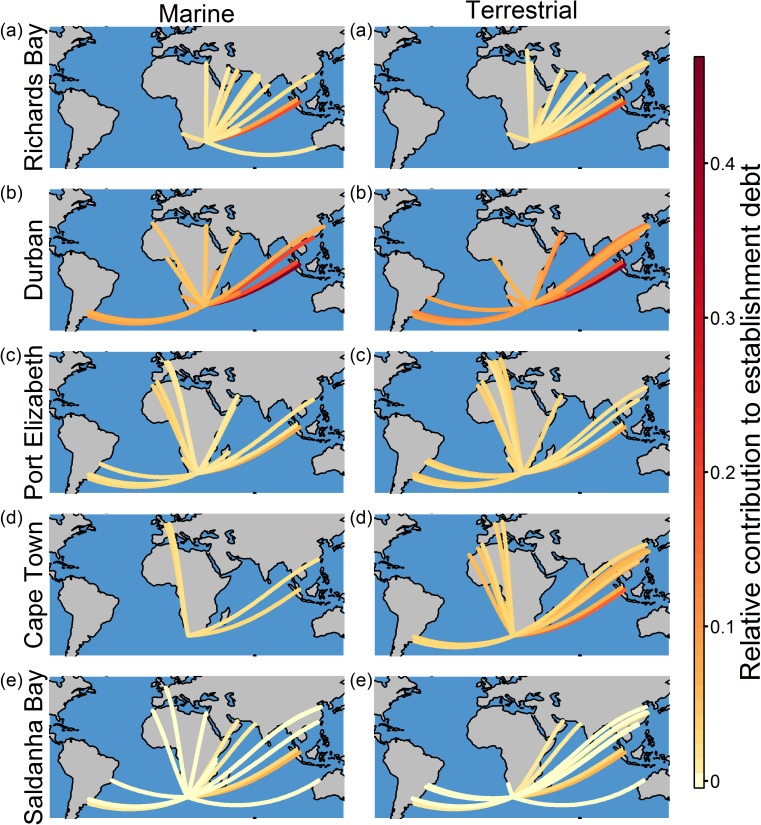
For each South African port, the twenty shipping routes from foreign ports with the highest relative contribution to marine and terrestrial establishment debt: (a) Richards Bay, (b) Durban, (c) Port Elizabeth, (d) Cape Town and (e) Saldanha Bay. The depicted routes are not the actual routes followed by ships.

There was significant variation across the South African ports in terms of the relative contribution of shipping routes to marine and terrestrial establishment debt, with the routes to Durban contributing the most (Fig 1, also see Fig 2). For all South African ports, few shipping routes from foreign ports contribute greatly to marine and terrestrial establishment debt, but many routes have a low contribution (Fig 1). For both environments, the route rankings for the South African ports were significantly different from one another (Kendall’s coefficient of concordance marine environment: *W* = 0.07, *χ*^*2*^ = 80.56, *P* = 1.00; terrestrial environment: *W* = 0.06, *χ*^*2*^ = 102.64, *P* = 1.00; also see Fig 2). For some South African ports (e.g. Richards Bay and Durban), shipping routes from Asian ports (e.g. Port Klang (Malaysia) and Hong Kong (China)) contribute greatly to marine and terrestrial establishment debt, with the route from Singapore consistently making the greatest contribution (Fig 2). Amongst the shipping routes that have a high relative contribution to marine and terrestrial establishment debt, routes from Asian and African ports (e.g. Maputo (Mozambique) and Port Louis (Mauritius)) dominate for all South African ports, while for some South African ports routes from South America (e.g. Montevideo (Uruguay)), Europe (e.g. Algeciras and Las Palmas (both Spain)) and Australia (e.g. Bunbury and Port Hedland) also feature (Fig 2). Although the number of days travelled showed very little variation across the South African ports, inter-port variation in the relative contribution of the shipping routes to marine and terrestrial establishment debt might be largely attributed to variation across the ports in the number of ship visits and marine and terrestrial environmental distance (S6 Fig).

**Fig 3:**
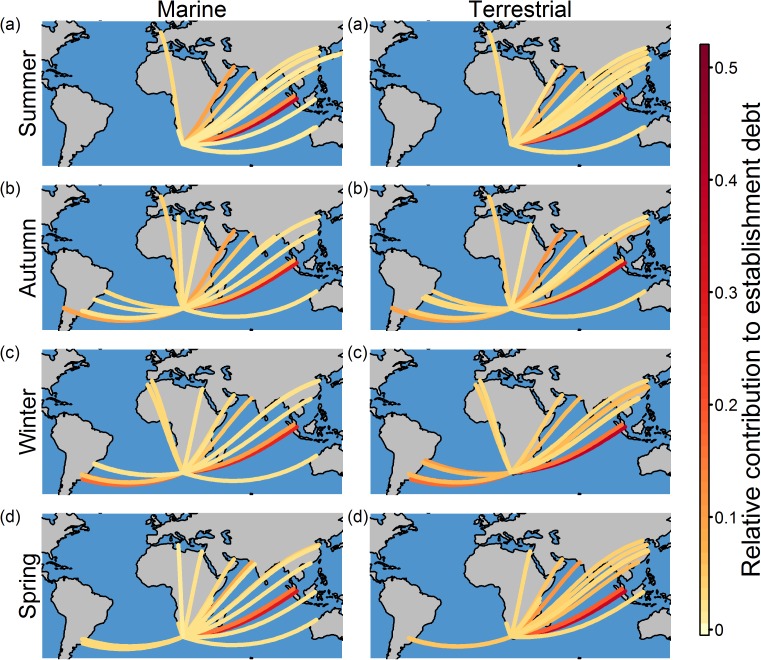
The twenty shipping routes from foreign ports, for each season, with the highest relative contribution to the marine and terrestrial establishment debt of Saldanha Bay. Seasons are based on those of the southern hemisphere: (a) summer, (b) autumn, (c) winter and (d) spring. The depicted routes are not the actual routes followed by ships.

Our preliminary seasonal analysis showed that the relative contribution of shipping routes from foreign ports to the marine and terrestrial establishment debt of the South African ports did not vary significantly across the seasons (Table 2 and S7 Fig). However for each South African port, the seasonal route rankings were significantly different from one another (Table 3). For example, although in autumn, winter and spring shipping routes from South America were amongst the twenty routes with the highest relative contribution to the marine and terrestrial establishment debt of Saldanha Bay, this is not the case in summer (Fig 3). For all South African ports there was little seasonal variation in the average number of days travelled by arriving ships, but the number of ship visits and the marine and terrestrial environmental distances varied seasonally (S8 Fig).

**Table 2:** Results of Kruskal-Wallis tests testing for seasonal variation in the relative contribution of shipping routes from foreign ports to the marine and terrestrial establishment debt of the South African ports.

Environment	South African port	*χ*^*2*^	df	*P*
Marine	Richards Bay	0.51	3	0.92
	Durban	2.78	3	0.43
	Port Elizabeth	1.55	3	0.67
	Cape Town	1.52	3	0.68
	Saldanha Bay	3.04	3	0.39
Terrestrial	Richards Bay	1.83	3	0.61
	Durban	5.67	3	0.13
	Port Elizabeth	5.45	3	0.14
	Cape Town	2.31	3	0.51
	Saldanha Bay	1.78	3	0.62

**Table 3:** Seasonal concordance for each South African port. A significant result would indicate that the route ranking for at least one season is concordant with another, but as none were significant, the seasonal route rankings for all South African ports are not concordant with one another.

Environment	South African port	W	*χ*^*2*^	*P*
Marine	Richards Bay	0.05	27.91	1.00
	Durban	0.06	76.79	1.00
	Port Elizabeth	0.04	19.77	1.00
	Cape Town	0.12	59.14	1.00
	Saldanha Bay	0.16	44.65	1.00
Terrestrial	Richards Bay	0.06	57.14	1.00
	Durban	0.07	125.53	1.00
	Port Elizabeth	0.04	29.12	1.00
	Cape Town	0.09	66.47	1.00
	Saldanha Bay	0.11	46.89	1.00

### Comparison with field data

**Fig 4:**
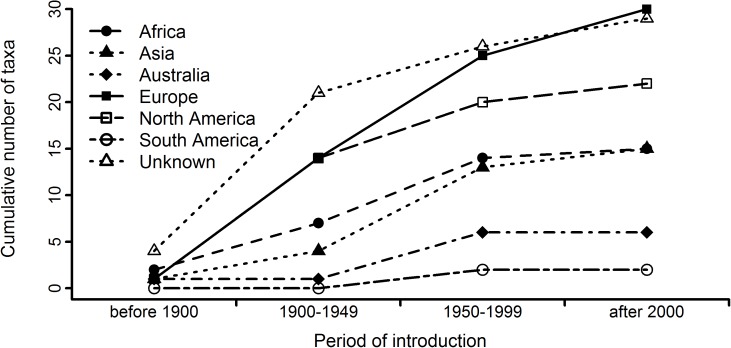
Temporal changes in the region of origin of marine species introduced to South Africa. Data were obtained from the dataset presented in Faulkner et al. [68]. Not included are species for which date of introduction data were not available (n = 17).

According to field data from South African catalogues, 100 marine species have been introduced to South Africa through biofouling or ballast water. Region of origin data were available for 61 of these species, with region of origin and date of introduction data available for 54 species (Fig 4). The field data do not correspond with our results. Our results indicate that a greater number of marine species should have been introduced to the biogeographical region associated with Durban (sub-tropical east coast) than that associated with Saldanha Bay (cool-temperate west coast). However, more introduced species have been recorded along the cool-temperate west coast than the sub-tropical east coast [69]. Our results also suggest that many of South Africa’s introduced marine species should originate from Asia and Africa. However, most introduced organisms originate from regions like Europe; a pattern that holds whether all introductions or only recent (after 2000) introductions are considered (Fig 4).

### Watch list species associated with high risk shipping routes

**Fig 5:**
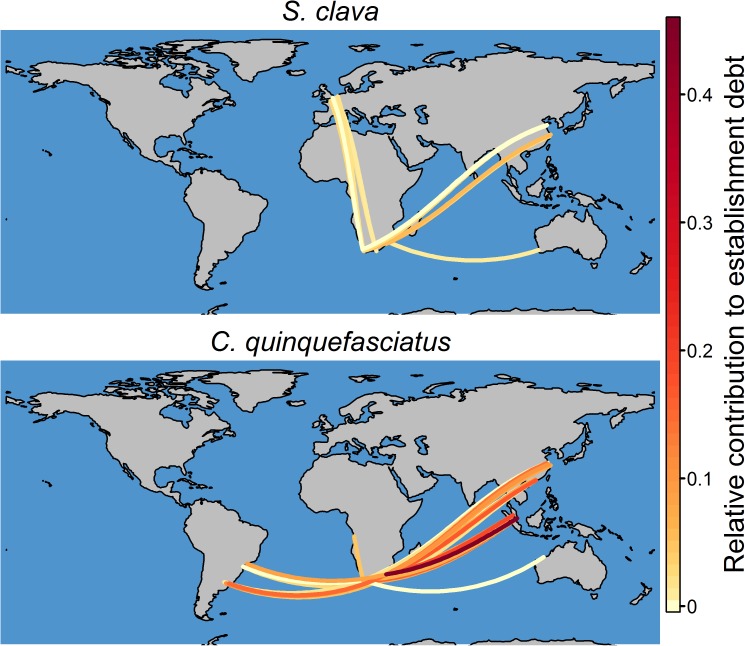
The high risk shipping routes which may facilitate the introduction of the marine tunicate *Styela clava* and the mosquito *Culex quinquefasciatus* to South African ports. The depicted routes are not the actual routes followed by ships.

Of the watch list species, 33 marine species and 292 terrestrial species occur in the Bailey ecoregion divisions and Köppen-Geiger climate zones that are associated with the source ports of high risk routes (i.e. the 20 routes for each South African port with the highest relative contribution to establishment debt (see materials and methods)) (S2 Table). Based on data obtained from the Global Invasive Species Database, shipping has previously facilitated the introduction of 23 of these marine species and 31 of these terrestrial species (S2 Table). However, pathway of introduction data were not available for three marine species and 100 terrestrial species (S2 Table). Based on the available information, of the watch list species ~20 marine species and between 10 and 28 terrestrial species are most likely to be introduced to the South African ports through high risk shipping routes (S3 Table). Different watch list species are, however, likely to be introduced to South African ports through different routes. For example, routes from China, Europe and Australia might facilitate the introduction of the marine tunicate *Styela clava*, while the mosquito *Culex quinquefasciatus* might be introduced through many routes from Asia (including that from Singapore), Africa, South America and Australia (Fig 5). As the source ports are associated with different numbers of watch list species, some high risk shipping routes might facilitate the introduction of more watch list species than others (S9 Fig). Furthermore, of the high risk routes, those with a high relative contribution to establishment debt are not necessarily associated with a relatively high number of watch list species. For example, in comparison to routes from Singapore, the relative contribution of Chinese and European shipping routes to marine and terrestrial establishment debt is lower, but these routes are associated with a higher number of watch list species than those from Singapore (Fig 2 and S9 Fig).

## Discussion

### Contribution of shipping to South Africa’s establishment debt

Models have demonstrated that, globally, the risk of shipping-facilitated invasions varies both geographically and temporally [10,35,39]. Our analysis similarly showed that the relative contribution of shipping routes to marine and terrestrial establishment debt varies across South African ports, with routes to Durban having the highest, relative contribution. For marine invasions facilitated by ballast water, Durban is the South African port with the highest invasion probability [35] and the Durban-Singapore route, the shipping route with the highest risk for South Africa (for both marine and terrestrial environments), is one of the main routes globally [34]. Finally, mosquitos that are often transported by ships [74] have been intercepted at Durban numerous times [75].

For some ports around the globe there are few shipping routes which are likely to facilitate invasions, while for others there are a wide variety of routes that pose a risk [10,35]. All South African ports have only a few high risk connections, however, these shipping routes vary across the ports. For example, many ships travel along a few routes from Australia to Richards Bay and Saldanha Bay and thus, for these ports, these routes have a high relative contribution to terrestrial and/or marine establishment debt, however, this is not the case for Durban, Port Elizabeth and Cape Town. South Africa is not unique in this aspect, and inter-port variations in the source regions of ships have been demonstrated for other regions (e.g. the USA [50]).

Shipping routes from Asian ports have a high relative contribution to South Africa’s establishment debt. The high potential for alien species to spread from Asia to South Africa is not surprising, as previous research has demonstrated that South African ports are in the same large container ship and bulk dry carrier ship communities as Asian ports [11]. Port communities are groups of ports that have many links with each other but few links with other groups [11]. As a consequence, the spread of non-native species between ports in the same community will be greater than that between ports in different communities [11].

Theoretical studies have suggested that, due to seasonal variations in shipping intensity and environmental conditions, the risk of shipping-facilitated marine invasions varies seasonally [35]. Similarly, for terrestrial species that are often transported by ships, like the gypsy moth (*Lymantia dispar*), phenology models have shown that the probability of establishment given introduction varies within the year, partly due to the mismatch of seasons between source and destination ports (e.g. summer at the source port but winter at the destination port) [39]. Although there was some seasonal variation in the relative contribution of the shipping routes to the establishment debt of South African ports, this variation was not significant. However, and despite this, for each South African port the relative importance of the shipping routes (i.e. the route rankings) varied significantly across the seasons. To determine if this variation is consistent across years an analysis of at least three years of data is required. A multi-year comparison was not done here due to the cost of ship movement data; however, such an analysis would better clarify the risk of invasion posed to South Africa and the consequences of seasonal variation for prevention strategies could be explored further. Furthermore, although our analysis was not species-specific and thus did not consider phenology, it is important to note that phenology undoubtedly plays an important role in determining seasonal variation in the transportation, introduction and establishment of alien species [39].

### Comparison with field data

Interception data are not readily available, and so previous research has used field data on the number of observed alien species to validate results [e.g. 35]. Although it could be argued that recent shipping data are unlikely to predict introductions and invasions that have already occurred, models based on recent shipping data have produced global predictions that agree with the number of observed marine alien species [35], and have successfully simulated the historical spread of these organisms [76]. As Durban has been South Africa’s most important port since the beginning of the 20^th^ century [77], we expected Durban to have the highest number of introduced species. However, this was not the case. This might be due to biases in sampling effort. South Africa’s east coast has been poorly surveyed for marine species in comparison to the west coast [20] (e.g. the Global Ballast Water Management Programme, GloBallast, sampled Saldanha Bay on the west coast but not Durban on the east coast [78]). The disagreement between our results and field data might also be due to recent changes in South Africa’s major trading partners. Since 2000 Asian countries have become more important and since 2010 these countries have dominated South African imports [46; also see S10 Fig]. Although there is a time-lag between the introduction of an organism, when it is discovered and when it becomes invasive (i.e. invasion debt) [40,47], we expected that recent introductions would tend to originate from Asia. This was not the case, and recently detected marine alien species in South Africa tend to originate from Europe (the historical major trade partner (S10 Fig)) rather than from Asia (the current major trade partner). It may take time before patterns of introduction reflect the patterns of colonisation pressure identified here.

### Watch list species associated with high risk shipping routes

Of the high risk routes (i.e. the 20 routes, for each South African port, with the highest relative contribution to establishment debt), some were associated with more marine and terrestrial watch list species than others, with a few of the routes from China and Europe being associated with a particularly high number of species. Although these patterns might reflect differences in the invasive species pools associated with different ports of origin, GBIF data are geographically biased (developed countries have higher numbers of records than developing countries). Therefore, these results might alternatively reflect biases driven by geographical variations in factors like sampling [79,80]. Additionally, it is important to note that the South African watch list does not contain all species that might pose an invasion threat in the future (e.g. those that currently have no history of invasion), and that pathway of introduction data were not available from the Global Invasive Species Database for some of the watch list species. As such, the high risk routes identified here are likely to transport other alien species that have the potential to become invasive.

### Management implications

Variation across South African ports in terms of the relative contribution of shipping routes to establishment debt and in the ranking of the shipping routes means that port-specific prevention strategies need to be developed. However, for all South African ports, ships from Singapore and a few other high risk foreign ports must be targeted for inspections (e.g. of cargo) and assessments (e.g. of on-board ballast water management). Additionally, resources available for the prevention of invasions can be allocated to the South African ports based on the relative contribution of the shipping routes to establishment debt, with Durban receiving the greatest portion of the resources. For the South African ports there appeared to be limited seasonal variation in the relative contribution of the shipping routes to establishment debt, thus potentially negating the need for season-specific resource allocation (i.e. resources should be consistent across the seasons). However, for each South African port, the rankings of the shipping routes did vary across the seasons. Thus prevention strategies for shipping in South Africa might need to consider the seasons, by targeting ships from foreign ports at times of the year when they pose a particularly high risk. For example, our preliminary results showed that ships from South America (e.g. Montevideo (Uruguay) and La Plata (Argentina)) have a relatively high contribution to the establishment debt of Saldanha Bay in autumn, winter and spring, however, this is not the case in summer, implying that ship routes could be seasonally prioritised for inspections or assessments. By combining the establishment debt results with data on watch list species, our results might also inform surveillance by providing an indication of particular shipping routes that should be monitored for the introduction of particular species. Furthermore, surveys for new incursions should focus on the ports where introductions and invasions are most likely to occur (i.e. Durban).

## Supporting information

S1 Fig The location of the five selected South African ports, and the benthic bioregions as per Sink et al. [70]. (DOCX)

S2 Fig The number of ocean going vessels arriving at South African ports each year. (DOCX)

S3 Fig Examples of pairwise-comparisons of the climatic conditions of ports. (DOCX)

S4 Fig Examples of pairwise-comparisons of the marine environmental conditions of ports. (DOCX)

S5 Fig The biogeographical regions as classified by Mead et al. [69] and the position of the five selected South African ports. (DOCX)

S6 Fig The number of ship visits, number of days travelled, marine environmental distance and terrestrial environmental distance for the South African ports. (DOCX)

S7 Fig The seasonal, relative contribution of shipping routes from foreign ports to the marine and terrestrial establishment debt of South African ports. (DOCX)

S8 Fig Seasonal variation in the number of ship visits, number of days travelled, marine environmental distance and terrestrial environmental distance of the South African ports. (DOCX)

S9 Fig The number of marine and terrestrial watch list species that might be transported along the twenty shipping routes to each South African port with the highest relative contribution to marine and terrestrial establishment debt. (DOCX)

S10 Fig Temporal trends in the contribution of different regions to South African merchandise imports. (DOCX)

S1 Table Details of the foreign ports included in the analysis. (DOCX)

S2 Table Species on the watch list developed for South Africa by Faulkner et al. [71], the environment in which they occur (marine or terrestrial), whether they are found in climate zones or marine ecoregions that are associated with the source ports of high risk shipping routes, and if so whether they have a history of shipping-facilitated introduction. (DOCX)

S3 Table Marine and terrestrial watch list species that might be transported along the twenty shipping routes to each South African port with the highest relative contribution to marine and terrestrial establishment debt. (DOCX)

S4 Table The relative contribution of shipping routes from foreign ports to marine and terrestrial establishment debt. (XLSX)

S5 Table The seasonal, relative contribution of shipping routes from foreign ports to the marine and terrestrial establishment debt of South African ports. (XLSX)
