# Supplementary material for: Prioritising surveillance for alien organisms transported as stowaways on ships travelling to South Africa
Source: PLoS One. 2017 Apr 5;12(4):e0173340. doi: 10.1371/journal.pone.0173340 (PMC5381868; doi:10.1371/journal.pone.0173340)
Supplement: S1 Fig — (DOCX) [file pone.0173340.s001.docx]

**
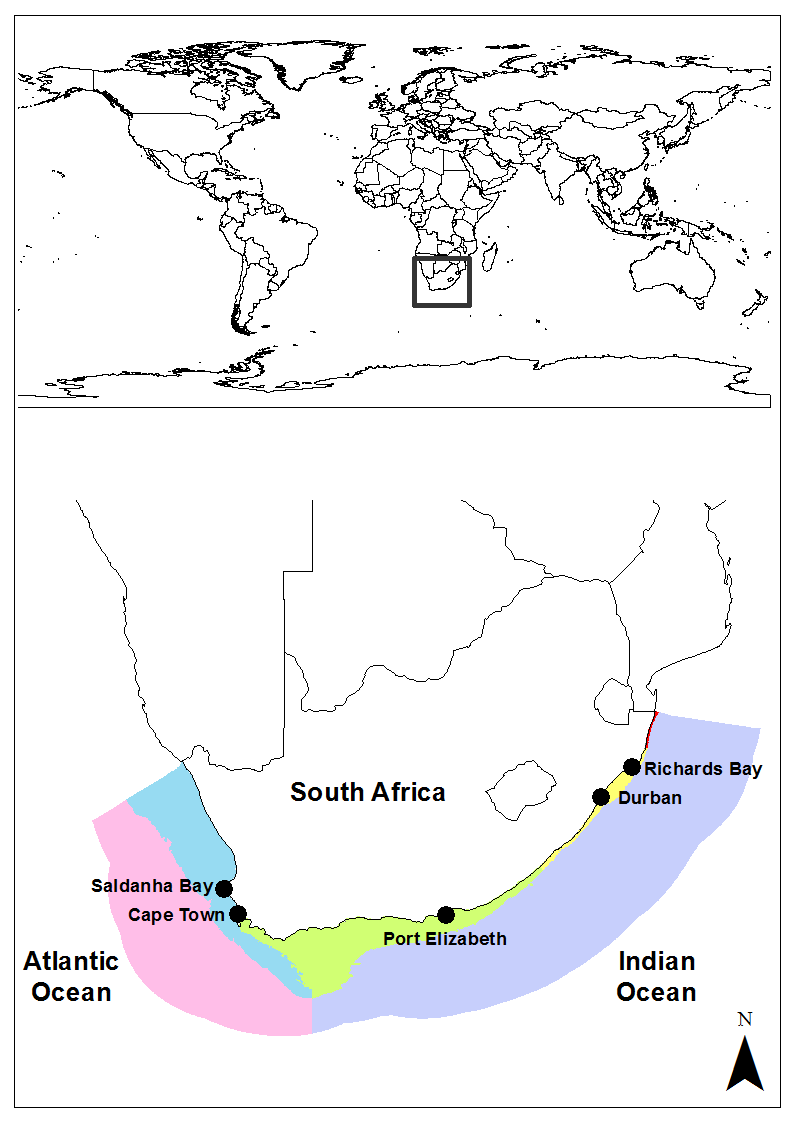
**

S1 Fig. The location of the five selected South African ports, and the benthic bioregions as per Sink et al. (2011). The Delagoa (red), Natal (yellow), Agulhas (green) and Southern Benguela (blue) bioregions include the coast, continental shelves and shelf break, whereas the Southwest Indian (purple) and Southeast Atlantic (pink) bioregions are offshore and include the slope and upper basins.
