# Supplementary material for: Prioritising surveillance for alien organisms transported as stowaways on ships travelling to South Africa
Source: PLoS One. 2017 Apr 5;12(4):e0173340. doi: 10.1371/journal.pone.0173340 (PMC5381868; doi:10.1371/journal.pone.0173340)
Supplement: S2 Fig — (DOCX) [file pone.0173340.s002.docx]

S2 Fig. The number of ocean going vessels arriving at South African ports (arranged from east to west) each year (2003 – 2008). These data were obtained from the National Ports Authority of South Africa.
