# Supplementary material for: Prioritising surveillance for alien organisms transported as stowaways on ships travelling to South Africa
Source: PLoS One. 2017 Apr 5;12(4):e0173340. doi: 10.1371/journal.pone.0173340 (PMC5381868; doi:10.1371/journal.pone.0173340)
Supplement: S3 Fig — (DOCX) [file pone.0173340.s003.docx]

Fig A. Monthly precipitation and temperature data for Brisbane (Australia) and Durban (South Africa), and the monthly environmental distance (measured using Euclidean distance) between the two ports.

Fig B. Monthly precipitation and temperature data for Singapore and Durban (South Africa), and the monthly environmental distance (measured using Euclidean distance) between the two ports.

Fig C. Monthly precipitation and temperature data for Buenaventura (Colombia) and Durban (South Africa), and the monthly environmental distance (measured using Euclidean distance) between the two ports.
