# Supplementary material for: Prioritising surveillance for alien organisms transported as stowaways on ships travelling to South Africa
Source: PLoS One. 2017 Apr 5;12(4):e0173340. doi: 10.1371/journal.pone.0173340 (PMC5381868; doi:10.1371/journal.pone.0173340)
Supplement: S4 Fig — (DOCX) [file pone.0173340.s004.docx]

Fig A. Monthly sea surface salinity (black lines) and temperature data (red lines) for Mackay (Australia) and Durban (South Africa), and the monthly environmental distance (measured using Euclidean distance) between the two ports.

Fig B. Monthly sea surface salinity (black lines) and temperature data (red lines) for Singapore and Durban (South Africa), and the monthly environmental distance (measured using Euclidean distance) between the two ports.

Fig C. Monthly sea surface salinity (black lines) and temperature data (red lines) for Lulea (Sweden) and Durban (South Africa), and the monthly environmental distance (measured using Euclidean distance) between the two ports.
