# Supplementary material for: Prioritising surveillance for alien organisms transported as stowaways on ships travelling to South Africa
Source: PLoS One. 2017 Apr 5;12(4):e0173340. doi: 10.1371/journal.pone.0173340 (PMC5381868; doi:10.1371/journal.pone.0173340)
Supplement: S5 Fig — (DOCX) [file pone.0173340.s005.docx]

**
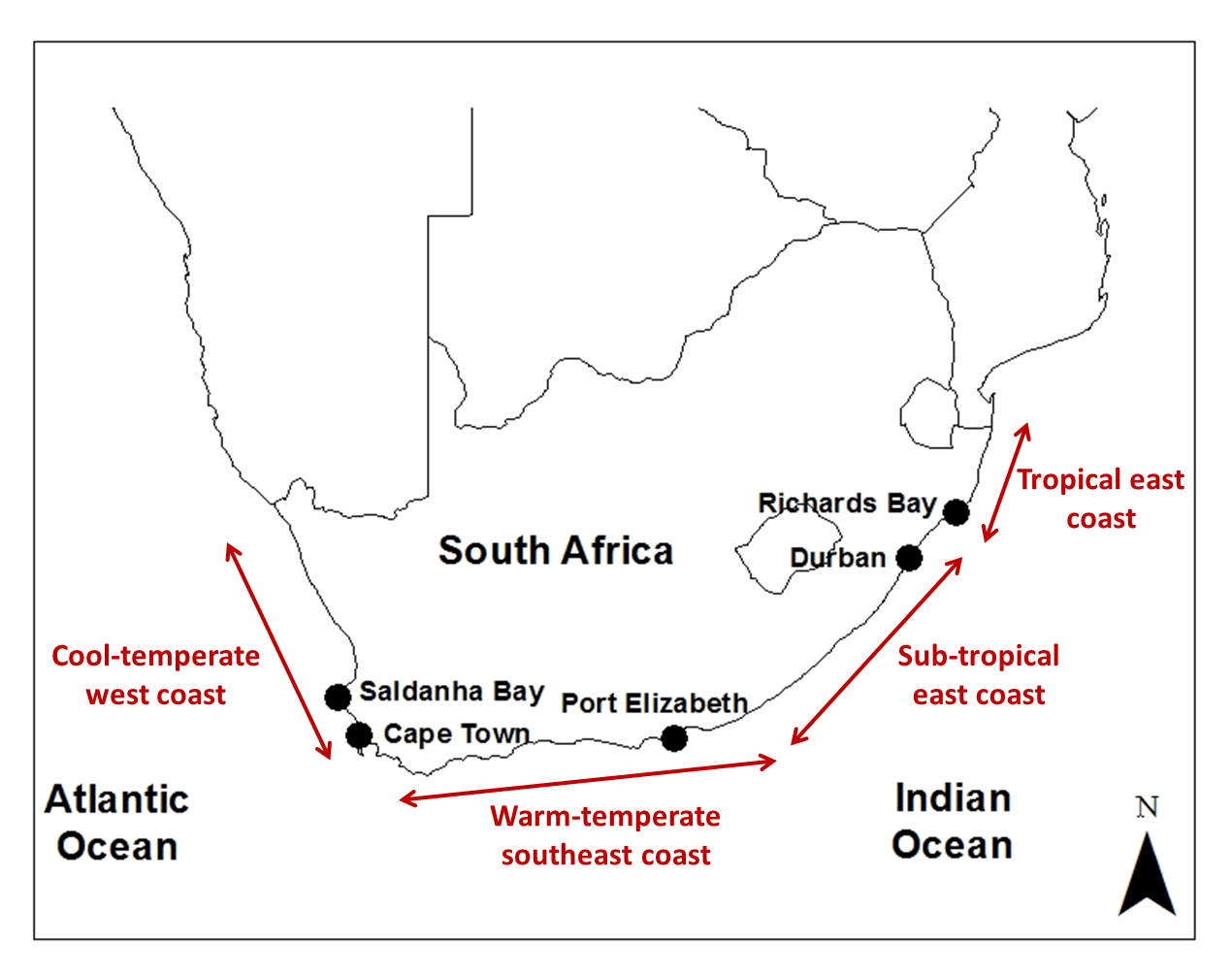
**

S5 Fig. The biogeographical regions (red) as classified by Mead et al. (2011) and the position of the five selected South African ports.
