# Supplementary material for: Prioritising surveillance for alien organisms transported as stowaways on ships travelling to South Africa
Source: PLoS One. 2017 Apr 5;12(4):e0173340. doi: 10.1371/journal.pone.0173340 (PMC5381868; doi:10.1371/journal.pone.0173340)
Supplement: S6 Fig — (DOCX) [file pone.0173340.s006.docx]

S6 Fig. The (a) number of ship visits, (b) number of days travelled, (c) marine environmental distance and (d) terrestrial environmental distance for the South African ports. Boxplots represent median and interquartile range.
