# Supplementary material for: Prioritising surveillance for alien organisms transported as stowaways on ships travelling to South Africa
Source: PLoS One. 2017 Apr 5;12(4):e0173340. doi: 10.1371/journal.pone.0173340 (PMC5381868; doi:10.1371/journal.pone.0173340)
Supplement: S7 Fig — (DOCX) [file pone.0173340.s007.docx]

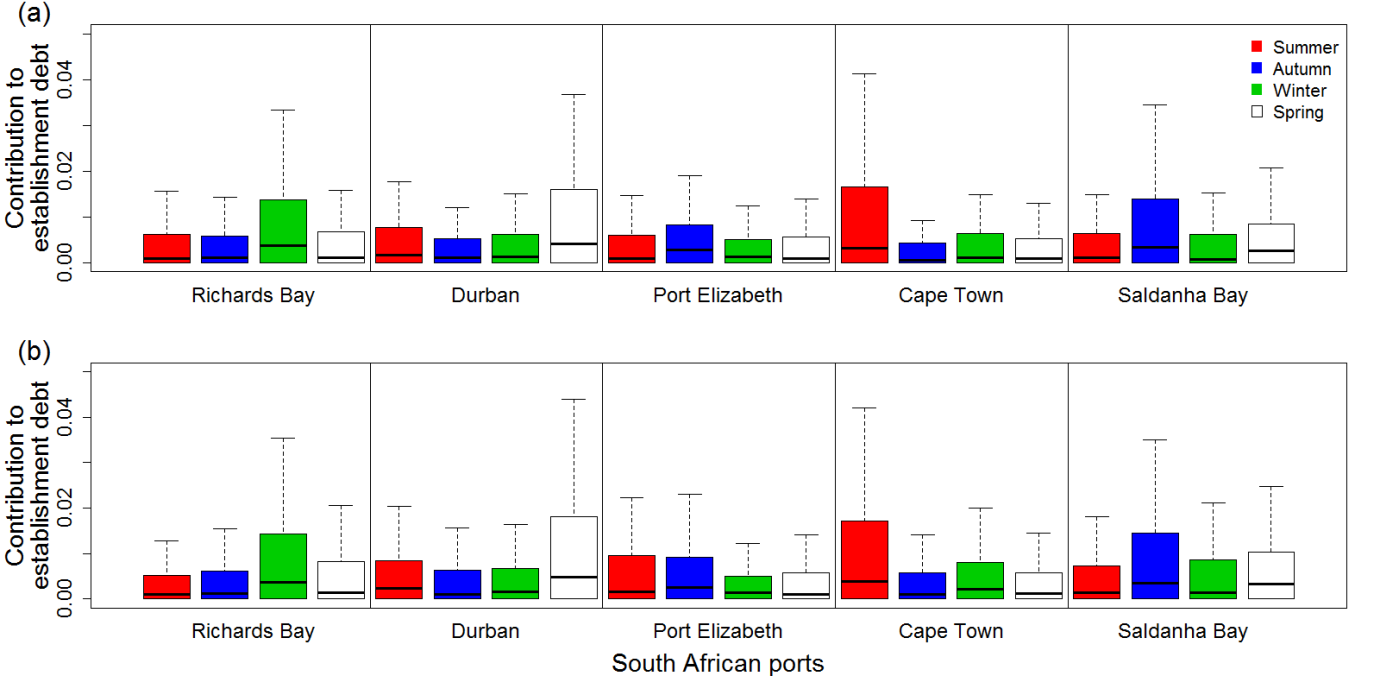


S7 Fig. The seasonal, relative contribution of shipping routes from foreign ports to the (a) marine and (b) terrestrial establishment debt of South African ports. Boxplots represent median and interquartile range. Seasons are based on those of the southern hemisphere.
