# Supplementary material for: Prioritising surveillance for alien organisms transported as stowaways on ships travelling to South Africa
Source: PLoS One. 2017 Apr 5;12(4):e0173340. doi: 10.1371/journal.pone.0173340 (PMC5381868; doi:10.1371/journal.pone.0173340)
Supplement: S8 Fig — (DOCX) [file pone.0173340.s008.docx]

S8 Fig. Seasonal variation in the (a) number of ship visits, (b) number of days travelled, (c) marine environmental distance and (d) terrestrial environmental distance of the South African ports. Seasons are based on those of the southern hemisphere: summer is in red, autumn is in blue, winter is in green and spring is in white. Boxplots represent median and interquartile range.
