# Supplementary material for: Prioritising surveillance for alien organisms transported as stowaways on ships travelling to South Africa
Source: PLoS One. 2017 Apr 5;12(4):e0173340. doi: 10.1371/journal.pone.0173340 (PMC5381868; doi:10.1371/journal.pone.0173340)
Supplement: S9 Fig — (DOCX) [file pone.0173340.s009.docx]

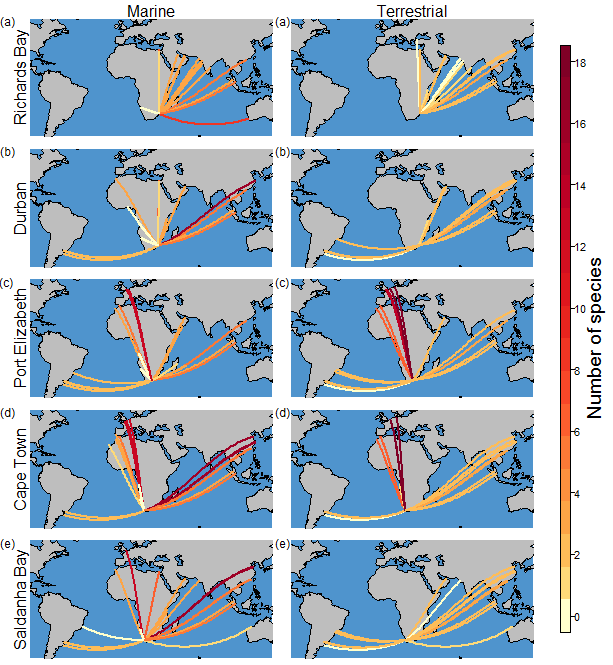


S9 Fig. The number of marine and terrestrial watch list species that might be transported along the twenty shipping routes to each South African port with the highest relative contribution to marine and terrestrial establishment debt: (a) Richards Bay, (b) Durban, (c) Port Elizabeth, (d) Cape Town and (e) Saldanha Bay. Routes that are associated with no watch list species are not shown. The depicted routes are not the actual routes followed by ships.
