# Supplementary material for: Prioritising surveillance for alien organisms transported as stowaways on ships travelling to South Africa
Source: PLoS One. 2017 Apr 5;12(4):e0173340. doi: 10.1371/journal.pone.0173340 (PMC5381868; doi:10.1371/journal.pone.0173340)
Supplement: S10 Fig — (DOCX) [file pone.0173340.s010.docx]

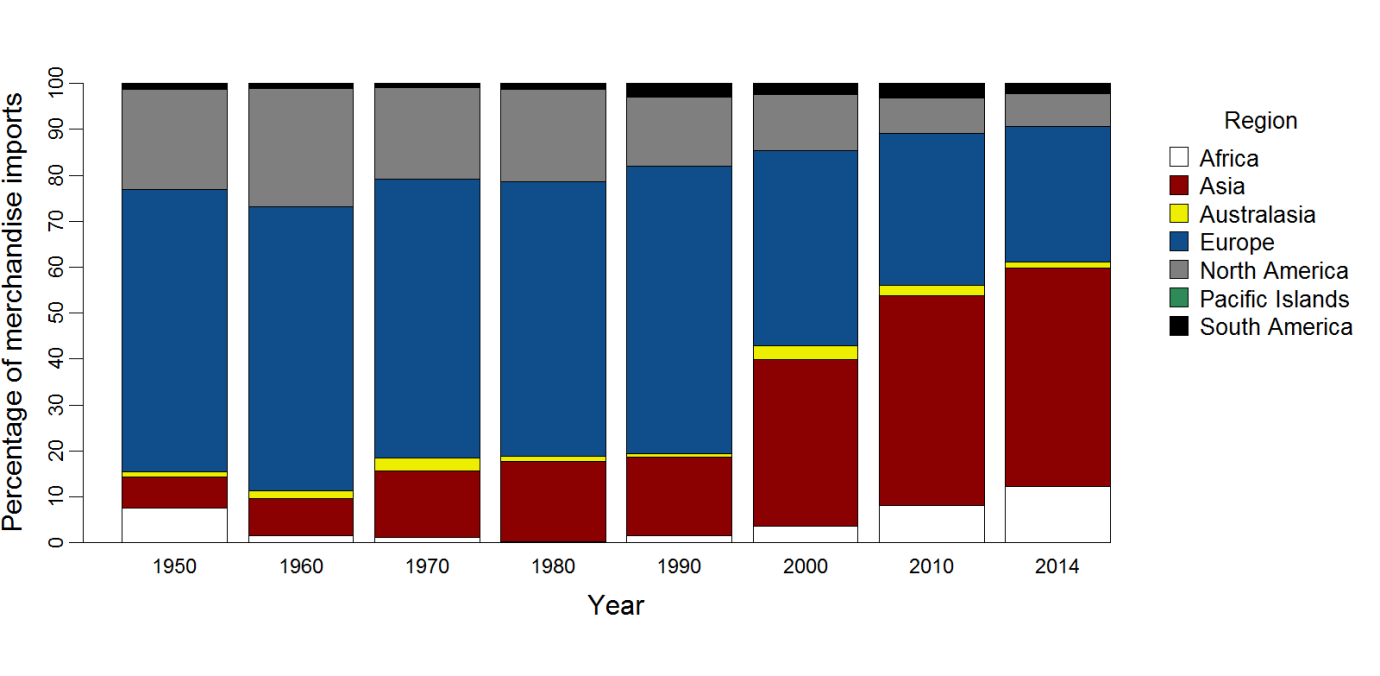


S10 Fig. Direction of trade statistics from the International Monetary Fund showing temporal trends in the contribution of different regions to South African merchandise imports.
