# Supplementary material for: Prioritising surveillance for alien organisms transported as stowaways on ships travelling to South Africa
Source: PLoS One. 2017 Apr 5;12(4):e0173340. doi: 10.1371/journal.pone.0173340 (PMC5381868; doi:10.1371/journal.pone.0173340)
Supplement: S1 Table — (DOCX) [file pone.0173340.s011.docx]

S1 Table. Details of the foreign ports included in the analysis. The ports are ordered alphabetically by country.

| Port | Country | Latitude | Longitude |
| --- | --- | --- | --- |
| Durres | Albania | 41.31306 | 19.45111 |
| Annaba | Algeria | 36.89972 | 7.75667 |
| Arzew | Algeria | 35.86056 | -0.30972 |
| Bejaia | Algeria | 36.72167 | 5.07389 |
| Djen Djen | Algeria | 36.82083 | 5.77 |
| Mostaganem | Algeria | 35.93472 | 0.08083 |
| Oran | Algeria | 35.70583 | -0.64222 |
| Skikda | Algeria | 36.87778 | 6.94389 |
| Cabinda | Angola | -5.55139 | 12.19278 |
| Lobito | Angola | -12.35361 | 13.54361 |
| Luanda | Angola | -8.81389 | 13.23083 |
| Namibe | Angola | -15.16472 | 12.15778 |
| Porto Amboim | Angola | -10.72806 | 13.75667 |
| Porto Saco | Angola | -15.13333 | 12.13333 |
| Soyo | Angola | -6.12 | 12.28222 |
| Philipsburg | Anguilla | 18.03333 | -63.05 |
| Arroyo Seco | Argentina | -33.12917 | -60.51778 |
| Atucha | Argentina | -33.95694 | -59.25111 |
| Bahia Blanca | Argentina | -38.73722 | -62.28972 |
| Buenos Aires | Argentina | -34.59028 | -58.3775 |
| Campana | Argentina | -34.15667 | -58.96639 |
| Comodoro Rivadavia | Argentina | -45.85694 | -67.48222 |
| Concepcion del Uruguay | Argentina | -32.47944 | -58.22833 |
| Escobar | Argentina | -34.35 | -58.76667 |
| La Plata | Argentina | -34.8725 | -57.90694 |
| Mar del Plata | Argentina | -38.03306 | -57.54472 |
| Puerto Deseado | Argentina | -47.75028 | -65.89417 |
| Puerto Madryn | Argentina | -42.73861 | -65.04722 |
| Punta Colorada | Argentina | -41.7 | -65.03333 |
| Ramallo | Argentina | -33.49722 | -60.01083 |
| Rio Gallegos | Argentina | -51.61694 | -69.22111 |
| Rosario | Argentina | -32.95528 | -60.635 |
| San Antonio Este | Argentina | -40.81694 | -64.75194 |
| Villa Constitucion | Argentina | -33.22361 | -60.33194 |
| Zarate | Argentina | -34.09111 | -59.02056 |
| Oranjestad | Aruba | 12.52194 | -70.03889 |
| San Nicolas Bay | Aruba | 12.43639 | -69.92306 |
| Abbot Point | Australia | -19.885 | 148.07806 |
| Adelaide | Australia | -34.85028 | 138.56694 |
| Botany Bay | Australia | -33.96528 | 151.22444 |
| Brisbane | Australia | -27.42222 | 153.11111 |
| Broome | Australia | -17.96194 | 122.23361 |
| Bunbury | Australia | -33.33583 | 115.65028 |
| Bundaberg | Australia | -24.80667 | 152.40028 |
| Burnie | Australia | -41.0575 | 145.905 |
| Cape Cuvier | Australia | -24.22611 | 113.39389 |
| Dampier | Australia | -20.6825 | 116.71389 |
| Darwin | Australia | -12.44944 | 130.83972 |
| Eden | Australia | -37.06722 | 149.90639 |
| Esperance | Australia | -33.87361 | 121.89194 |
| Fremantle | Australia | -32.05917 | 115.75083 |
| Geelong | Australia | -38.13444 | 144.34917 |
| Geraldton | Australia | -28.77861 | 114.58972 |
| Gladstone | Australia | -23.83639 | 151.23583 |
| Gove | Australia | -12.18806 | 136.6875 |
| Hay Point | Australia | -21.2875 | 149.28472 |
| Karumba | Australia | -17.48889 | 140.83722 |
| Klein Point | Australia | -34.91722 | 137.79278 |
| Kurnell | Australia | -34 | 151.23333 |
| Lucinda | Australia | -18.52583 | 146.33111 |
| Mackay | Australia | -21.10778 | 149.2175 |
| Melbourne | Australia | -37.83111 | 144.91778 |
| Milner Bay | Australia | -13.85778 | 136.42417 |
| Onslow | Australia | -21.63333 | 115.1 |
| Port Alma | Australia | -23.58278 | 150.85972 |
| Port Bonython | Australia | -32.98889 | 137.76722 |
| Port Giles | Australia | -35.0225 | 137.76028 |
| Port Hedland | Australia | -20.31556 | 118.58222 |
| Port Kembla | Australia | -34.465 | 150.88917 |
| Port Lincoln | Australia | -34.72444 | 135.86917 |
| Port Pirie | Australia | -33.16861 | 138.01083 |
| Port Walcott | Australia | -20.5975 | 117.17611 |
| Sydney | Australia | -33.8575 | 151.20639 |
| Thevenard | Australia | -32.14694 | 133.64667 |
| Townsville | Australia | -19.25583 | 146.82861 |
| Wallaroo | Australia | -33.92944 | 137.62472 |
| Weipa | Australia | -12.66417 | 141.87056 |
| Whyalla | Australia | -33.0375 | 137.58694 |
| Wyndham | Australia | -15.45194 | 128.10306 |
| Baku | Azerbaijan | 40.37833 | 49.84889 |
| Mina Sulman | Bahrain | 26.24111 | 50.59278 |
| Sitra | Bahrain | 26.15417 | 50.63167 |
| Chittagong | Bangladesh | 22.32194 | 91.81722 |
| Mongla | Bangladesh | 22.51861 | 89.58722 |
| Bridgetown | Barbados | 13.10306 | -59.62389 |
| Antwerp | Belgium | 51.24472 | 4.41139 |
| Bruges | Belgium | 51.22194 | 3.22083 |
| Ghent | Belgium | 51.08806 | 3.74528 |
| Liege | Belgium | 50.635 | 5.57 |
| Ostend | Belgium | 51.22972 | 2.9225 |
| Zeebrugge | Belgium | 51.32306 | 3.21583 |
| Belize City | Belize | 17.48722 | -88.20028 |
| Cotonou | Benin | 6.35083 | 2.42306 |
| Alumar | Brazil | -2.68333 | -44.36667 |
| Angra dos Reis | Brazil | -23.00611 | -44.31306 |
| Aratu | Brazil | -12.78333 | -38.48333 |
| Barcarena | Brazil | -1.48333 | -48.66667 |
| Belem | Brazil | -1.44056 | -48.49167 |
| Cabedelo | Brazil | -6.97167 | -34.8375 |
| Fortaleza | Brazil | -3.71806 | -38.47139 |
| Gebig | Brazil | -23.05 | -44.23333 |
| Ilheus | Brazil | -14.78917 | -39.03194 |
| Imbituba | Brazil | -28.23361 | -48.65278 |
| Itacoatiara | Brazil | -3.15 | -58.45 |
| Itajai | Brazil | -26.89944 | -48.67056 |
| Itaqui | Brazil | -2.56667 | -44.35 |
| Maceio | Brazil | -9.66667 | -35.73333 |
| Madre de Deus | Brazil | -12.75 | -38.61667 |
| Manaus | Brazil | -3.15 | -60.01667 |
| Munguba | Brazil | -1.08333 | -52.38333 |
| Paranagua | Brazil | -25.5 | -48.51667 |
| Parati | Brazil | -23.21667 | -44.71667 |
| Pecem | Brazil | -3.55 | -38.81667 |
| Ponta da Madeira | Brazil | -2.56667 | -44.38333 |
| Ponta do Ubu | Brazil | -20.78333 | -40.58333 |
| Porto Alegre | Brazil | -30.03333 | -51.21667 |
| Portocel | Brazil | -19.85 | -40.05 |
| Praia Mole | Brazil | -20.28333 | -40.23333 |
| Recife | Brazil | -8.06667 | -34.86667 |
| Rio de Janeiro | Brazil | -22.91667 | -43.2 |
| Salvador | Brazil | -12.96667 | -38.51667 |
| Santarem | Brazil | -2.41667 | -54.7 |
| Santos | Brazil | -23.93333 | -46.33333 |
| Sao Francisco do Sul | Brazil | -26.23333 | -48.63333 |
| Sao Sebastiao | Brazil | -23.8 | -45.38333 |
| Sepetiba | Brazil | -22.93333 | -43.83333 |
| Suape | Brazil | -8.4 | -34.95 |
| Tramandai | Brazil | -30 | -50.08333 |
| Trombetas | Brazil | -1.46667 | -56.38333 |
| Tubarao | Brazil | -20.28333 | -40.25 |
| Vila do Conde | Brazil | -1.55 | -48.75 |
| Vitoria | Brazil | -20.3 | -40.33333 |
| Muara Port | Brunei | 5.03333 | 115.08333 |
| Balchik | Bulgaria | 43.38333 | 28.18333 |
| Bourgas | Bulgaria | 42.5 | 27.48333 |
| Varna | Bulgaria | 43.2 | 27.91667 |
| Phnom-Penh | Cambodia | 11.6 | 104.9 |
| Bonaberi | Cameroon | 4.06667 | 9.68333 |
| Douala | Cameroon | 4.05 | 9.7 |
| Limboh Term. | Cameroon | 4 | 9.13333 |
| Argentia | Canada | 47.3 | -53.98333 |
| Bay Bulls | Canada | 47.3 | -52.73333 |
| Bayside | Canada | 45.16667 | -67.13333 |
| Belledune | Canada | 47.91667 | -65.85 |
| Come by Chance | Canada | 47.8 | -54.01667 |
| Comeau Bay | Canada | 49.21667 | -68.15 |
| Contrecoeur | Canada | 45.88333 | -73.2 |
| Courtright | Canada | 42.8 | -82.45 |
| Cowichan Bay | Canada | 48.75 | -123.6 |
| Eastern Passage | Canada | 44.6 | -63.48333 |
| Fraser River Port | Canada | 49.2 | -122.91667 |
| Goderich | Canada | 43.75 | -81.75 |
| Halifax | Canada | 44.63333 | -63.55 |
| Montreal | Canada | 45.5 | -73.55 |
| Nanaimo | Canada | 49.16667 | -123.93333 |
| Oshawa | Canada | 43.86667 | -78.83333 |
| Port Alfred | Canada | 48.33333 | -70.86667 |
| Port Cartier | Canada | 50.01667 | -66.86667 |
| Port Colborne | Canada | 42.86667 | -79.25 |
| Port Weller | Canada | 43.23333 | -79.21667 |
| Prescott | Canada | 44.71667 | -75.51667 |
| Prince Rupert | Canada | 54.31667 | -130.36667 |
| Quebec | Canada | 46.81667 | -71.2 |
| Roberts Bank | Canada | 49.01667 | -123.13333 |
| Seven Islands | Canada | 50.1 | -66.38333 |
| Sorel | Canada | 46.05 | -73.11667 |
| St. Catharines | Canada | 43.16667 | -79.26667 |
| Thorold | Canada | 43.08333 | -79.16667 |
| Three Rivers | Canada | 46.35 | -72.55 |
| Thunder Bay | Canada | 48.41667 | -89.21667 |
| Toronto | Canada | 43.63333 | -79.38333 |
| Valleyfield | Canada | 45.21667 | -74.08333 |
| Antofagasta | Chile | -23.63333 | -70.43333 |
| Arica | Chile | -18.48333 | -70.33333 |
| Caleta Barquito | Chile | -26.35 | -70.65 |
| Caleta Coloso | Chile | -23.75 | -70.46667 |
| Caleta Patillos | Chile | -20.75 | -70.2 |
| Chanaral | Chile | -26.35 | -70.63333 |
| Coquimbo | Chile | -29.95 | -71.35 |
| Coronel | Chile | -37.03333 | -73.16667 |
| Huasco | Chile | -28.45 | -71.23333 |
| Iquique | Chile | -20.2 | -70.16667 |
| Lirquen | Chile | -36.71667 | -72.98333 |
| Mejillones | Chile | -23.1 | -70.46667 |
| Puerto Montt | Chile | -41.46667 | -72.95 |
| Punta Arenas | Chile | -53.16667 | -70.9 |
| Quintero | Chile | -32.76667 | -71.5 |
| San Vicente | Chile | -36.73333 | -73.15 |
| Talcahuano | Chile | -36.68333 | -73.1 |
| Tocopilla | Chile | -22.08333 | -70.23333 |
| Valparaiso | Chile | -33.01667 | -71.63333 |
| Bayuquan | China | 40.26667 | 122.1 |
| Beihai | China | 21.45 | 109.05 |
| Beiliang | China | 38.96667 | 121.8 |
| Beilun | China | 29.93333 | 121.88333 |
| Beipei | China | 29.81667 | 106.43333 |
| Caojing | China | 30.76667 | 121.4 |
| Changde | China | 29.03333 | 111.66667 |
| Changshu | China | 31.63333 | 120.73333 |
| Changzhou | China | 23.06194 | 113.42083 |
| Chenglingji | China | 29.43333 | 113.13333 |
| Chiwan | China | 22.46667 | 113.88333 |
| Chongming | China | 31.61667 | 121.36667 |
| Chuansha | China | 31.2 | 121.68333 |
| Dafeng | China | 33.2 | 120.41667 |
| Dalian | China | 38.91667 | 121.68333 |
| Dongguan | China | 23.05 | 113.71667 |
| Dongying | China | 37.45 | 118.46667 |
| Ezhou | China | 30.4 | 114.81667 |
| Fangcheng | China | 21.75 | 108.35 |
| Fengxian | China | 30.91667 | 121.43333 |
| Fuling | China | 29.7 | 107.33333 |
| Fuzhou | China | 26.05 | 119.3 |
| Guangzhou | China | 23.1 | 113.23333 |
| Haikou | China | 20.01667 | 110.26667 |
| Hangzhou | China | 30.25 | 120.16667 |
| Hefei | China | 31.85 | 117.26667 |
| Hong Kong | China | 22.3 | 114.16667 |
| Huanghua | China | 38.36667 | 117.33333 |
| Huangpu | China | 23.0975 | 113.42417 |
| Huizhou | China | 22.7 | 114.56667 |
| Huludao | China | 40.71667 | 120.98333 |
| Humen | China | 22.8 | 113.66667 |
| Jiangyin | China | 31.91667 | 120.25 |
| Jingjiang | China | 32.01667 | 120.3 |
| Jinshan | China | 30.71667 | 121.31667 |
| Jinzhou | China | 40.75 | 121.1 |
| Laizhou | China | 37.41667 | 119.95 |
| Lanshan | China | 35.08333 | 119.35 |
| Lianyungang | China | 34.73333 | 119.45 |
| Longkou | China | 37.68333 | 120.3 |
| Luzhou | China | 28.88333 | 105.43333 |
| Ma'anshan | China | 31.81667 | 118.53333 |
| Mabianzhou | China | 22.66667 | 114.65 |
| Macau | China | 22.2 | 113.55 |
| Majishan | China | 30.68333 | 122.41667 |
| Maocaojie | China | 29.06667 | 112.3 |
| Mawan | China | 22.48333 | 113.86667 |
| Meizhou | China | 25.18333 | 119 |
| Nanchang | China | 28.66667 | 115.88333 |
| Nanhui | China | 31.05 | 121.75 |
| Nanjing | China | 32.05 | 118.78333 |
| Nansha | China | 22.83333 | 113.56667 |
| Nantong | China | 32 | 120.81667 |
| Ningbo | China | 29.86667 | 121.55 |
| Panyu | China | 22.9 | 113.3 |
| Penglai | China | 37.81667 | 120.71667 |
| Pinghu | China | 30.68333 | 121 |
| Qingdao | China | 36.08333 | 120.3 |
| Qinglan | China | 19.58333 | 110.88333 |
| Qinhuangdao | China | 39.9 | 119.6 |
| Qinzhou | China | 21.95 | 108.61667 |
| Rizhao | China | 35.48333 | 119.48333 |
| Rongqi | China | 22.68333 | 113.21667 |
| Saiqi | China | 26.93333 | 119.68333 |
| Sanbaimen | China | 23.53333 | 117.08333 |
| Shanghai | China | 31.25 | 121.5 |
| Shanhaiguan | China | 39.98639 | 119.82917 |
| Shantou | China | 23.33333 | 116.75 |
| Shekou | China | 22.48333 | 113.9 |
| Shidao | China | 36.88333 | 122.46667 |
| Shilong | China | 23.1 | 113.81667 |
| Shiqiao | China | 22.91667 | 113.35 |
| Shuidong | China | 21.48333 | 111.06667 |
| Songxia | China | 25.68333 | 119.58333 |
| Taicang | China | 31.45 | 121.08333 |
| Taixing | China | 32.15 | 120 |
| Taizhou | China | 32.41667 | 119.41667 |
| Tangshan | China | 39.21667 | 119.01667 |
| Tianjin | China | 39.1 | 117.16667 |
| Weihai | China | 37.5 | 122.15 |
| Weitou | China | 24.53333 | 118.56667 |
| Wuhan | China | 30.58333 | 114.31667 |
| Wuxue | China | 29.85 | 115.55 |
| Xiamen | China | 24.45 | 118.06667 |
| Xiangfan | China | 32 | 112.13333 |
| Xingang | China | 38.98333 | 117.75 |
| Xinhui | China | 22.51667 | 113.03333 |
| Xintang | China | 23.11667 | 113.58333 |
| Xiuyu | China | 25.21667 | 118.98333 |
| Yangjiang | China | 21.85 | 111.93333 |
| Yangpu | China | 19.7 | 109.33333 |
| Yangzhou | China | 32.36667 | 119.36667 |
| Yantai | China | 37.56667 | 121.43333 |
| Yantian | China | 22.59 | 114.26833 |
| Yizheng | China | 32.2 | 119.2 |
| Yutang | China | 21.93333 | 112.76667 |
| Zhangjiagang | China | 31.96667 | 120.4 |
| Zhangzhou | China | 24.41667 | 118.05 |
| Zhanjiang | China | 21.2 | 110.41667 |
| Zhapu | China | 30.58333 | 121.08333 |
| Zhenhai | China | 29.96667 | 121.7 |
| Zhenjiang | China | 32.21667 | 119.40722 |
| Zhongshan | China | 22.51667 | 113.35 |
| Barranquilla | Colombia | 10.96667 | -74.78333 |
| Buenaventura | Colombia | 3.9 | -77.08333 |
| Covenas | Colombia | 9.41667 | -75.68333 |
| Mamonal | Colombia | 10.31667 | -75.51667 |
| Pozos Colorados Term. | Colombia | 11.15 | -74.25 |
| Santa Marta | Colombia | 11.25 | -74.21667 |
| Turbo | Colombia | 8.06667 | -76.73333 |
| Moroni | Comoros | -11.7 | 43.25 |
| Mutsamudu | Comoros | -12.16667 | 44.4 |
| Banana | Congo, DRC | -6.01667 | 12.4 |
| Boma | Congo, DRC | -5.85 | 13.05 |
| Matadi | Congo, DRC | -5.81667 | 13.45 |
| Punta Morales | Costa Rica | 10 | -84.96667 |
| Abidjan | Cote d'Ivory | 5.3 | -4 |
| Mali Losinj | Croatia | 44.53333 | 14.46667 |
| Rijeka | Croatia | 45.31667 | 14.43333 |
| Split | Croatia | 43.5 | 16.45 |
| Havana | Cuba | 23.13333 | -82.35 |
| Puerto Padre | Cuba | 21.28333 | -76.53333 |
| Larnaca | Cyprus | 34.91667 | 33.63333 |
| Limassol | Cyprus | 34.65417 | 33.01028 |
| Vassiliko Bay | Cyprus | 34.7 | 33.33333 |
| Aabenraa | Denmark | 55.03333 | 9.43333 |
| Aarhus | Denmark | 56.15 | 10.21667 |
| Copenhagen | Denmark | 55.7 | 12.61667 |
| Elsinore | Denmark | 56.03333 | 12.61667 |
| Esbjerg | Denmark | 55.46667 | 8.43333 |
| Fredericia | Denmark | 55.56667 | 9.75 |
| Frederikshavn | Denmark | 57.43333 | 10.55 |
| Grenaa | Denmark | 56.41333 | 10.91306 |
| Kalundborg | Denmark | 55.68333 | 11.08333 |
| Lindo | Denmark | 55.46667 | 10.53333 |
| Nyborg | Denmark | 55.3 | 10.78333 |
| Skagen | Denmark | 57.71667 | 10.6 |
| Djibouti | Djibouti | 11.6 | 43.15 |
| Boca Chica | Dominican Republic | 18.45 | -69.58333 |
| Caucedo | Dominican Republic | 18.4 | -69.61667 |
| Rio Haina | Dominican Republic | 18.41667 | -70 |
| Santo Domingo | Dominican Republic | 18.46667 | -69.88333 |
| Esmeraldas | Ecuador | 0.96667 | -79.68333 |
| Guayaquil | Ecuador | -2.28333 | -79.91667 |
| Manta | Ecuador | -0.93333 | -80.71667 |
| Posorja | Ecuador | -2.76667 | -80.33333 |
| Abu Kir | Egypt | 31.3 | 30.06667 |
| Abu Zenima | Egypt | 29.03333 | 33.11667 |
| Adabiya | Egypt | 29.86667 | 32.46667 |
| Damietta | Egypt | 31.38333 | 31.8 |
| El Dekheila | Egypt | 31.15 | 29.8 |
| Hamrawein | Egypt | 26.25 | 34.2 |
| Port Said | Egypt | 31.25 | 32.3 |
| Sadat | Egypt | 29.8 | 32.45 |
| Safaga | Egypt | 26.73333 | 33.93333 |
| Sokhna | Egypt | 29.68333 | 32.36667 |
| Suez | Egypt | 29.96667 | 32.55 |
| Acajutla | El Salvador | 13.6 | -89.83333 |
| Bata | Equatorial Guinea | 1.86667 | 9.76667 |
| Luba | Equatorial Guinea | 3.46667 | 8.55 |
| Malabo | Equatorial Guinea | 3.75 | 8.78333 |
| Massawa | Eritrea | 15.61667 | 39.48333 |
| Muuga | Estonia | 59.5 | 24.96667 |
| Paldiski | Estonia | 59.35 | 24.05 |
| Sillamae | Estonia | 59.4 | 27.78333 |
| Tallinn | Estonia | 59.45 | 24.75 |
| Fuglafjordur | Faroe Is. | 62.25 | -6.81667 |
| Suva | Fiji | -18.13333 | 178.41667 |
| Kokkola | Finland | 63.83333 | 23.13333 |
| Kotka | Finland | 60.46667 | 26.95 |
| Naantali | Finland | 60.46667 | 22.01667 |
| Pori | Finland | 61.48333 | 21.8 |
| Raahe | Finland | 64.68333 | 24.48333 |
| Uusikaupunki | Finland | 60.8 | 21.4 |
| Antibes | France | 43.58333 | 7.13333 |
| Antifer | France | 49.66667 | 0.16667 |
| Bayonne | France | 43.5 | -1.48333 |
| Blaye | France | 45.11667 | -0.66667 |
| Bordeaux | France | 44.83333 | -0.56667 |
| Brest | France | 48.38333 | -4.48333 |
| Caen | France | 49.18333 | -0.35 |
| Cherbourg | France | 49.64111 | -1.61417 |
| Donges | France | 47.3 | -2.06667 |
| Dunkirk | France | 51.05 | 2.35 |
| Fos | France | 43.38333 | 4.85 |
| Honfleur | France | 49.41667 | 0.23333 |
| La Nouvelle | France | 43.01667 | 3.06667 |
| La Rochelle | France | 46.15 | -1.15 |
| Lavera | France | 43.38333 | 5 |
| Le Havre | France | 49.48333 | 0.11667 |
| Le Legue | France | 48.53333 | -2.71667 |
| Le Verdon | France | 45.55 | -1.08333 |
| Lorient | France | 47.75 | -3.36667 |
| Marseilles | France | 43.33333 | 5.35 |
| Montoir | France | 47.33333 | -2.13333 |
| Nantes | France | 47.23333 | -1.56667 |
| Paris | France | 48.86667 | 2.33333 |
| Petit Couronne | France | 49.36667 | 1 |
| Port de Bouc | France | 43.4 | 4.98333 |
| Port Jerome | France | 49.46667 | 0.53333 |
| Rouen | France | 49.48333 | 1.08333 |
| Sete | France | 43.4 | 3.7 |
| St. Louis du Rhone | France | 43.38333 | 4.81667 |
| St. Malo | France | 48.64944 | -2.02528 |
| St. Nazaire | France | 47.26667 | -2.2 |
| Strasbourg | France | 48.56667 | 7.7 |
| Toulon | France | 43.11667 | 5.91667 |
| Papeete | French Polynesia | -17.53333 | -149.58333 |
| Gamba Term. | Gabon | -2.78333 | 10 |
| Libreville | Gabon | 0.4 | 9.43333 |
| Mayumba | Gabon | -3.38333 | 10.63333 |
| Owendo | Gabon | 0.28333 | 9.5 |
| Port Gentil | Gabon | -0.71667 | 8.78333 |
| Batumi | Georgia | 41.65 | 41.65 |
| Poti | Georgia | 42.15 | 41.65 |
| Blexen | Germany | 53.53333 | 8.53333 |
| Brake | Germany | 53.33333 | 8.48333 |
| Bremen | Germany | 53.08333 | 8.78333 |
| Bremerhaven | Germany | 53.55 | 8.58333 |
| Brunsbuttel | Germany | 53.9 | 9.13333 |
| Cuxhaven | Germany | 53.86667 | 8.7 |
| Emden | Germany | 53.35 | 7.18333 |
| Freiburg | Germany | 53.81667 | 9.3 |
| Hamburg | Germany | 53.55 | 9.96667 |
| Holtenau | Germany | 54.36667 | 10.15 |
| Karlsruhe | Germany | 49.05 | 8.33333 |
| Kiel | Germany | 54.31667 | 10.13333 |
| Krefeld | Germany | 51.33333 | 6.56667 |
| Neuhaus | Germany | 53.8 | 9.03333 |
| Nordenham | Germany | 53.48333 | 8.48333 |
| Rendsburg | Germany | 54.31667 | 9.66667 |
| Rostock | Germany | 54.15 | 12.1 |
| Weissenthurm | Germany | 50.41667 | 7.48333 |
| Wilhelmshaven | Germany | 53.51667 | 8.15 |
| Wismar | Germany | 53.9 | 11.46667 |
| Takoradi | Ghana | 4.88333 | -1.73333 |
| Tema | Ghana | 5.61667 | 0.01667 |
| Gibraltar | Gibraltar | 36.15 | -5.33333 |
| Agioi Theodoroi | Greece | 37.9 | 23.05 |
| Aliveri | Greece | 38.38333 | 24.05 |
| Aspropyrgos | Greece | 38.01667 | 23.58333 |
| Drepanon | Greece | 38.78333 | 20.73333 |
| Eleusis | Greece | 38.03333 | 23.5 |
| Kalamata | Greece | 37 | 22.11667 |
| Katakolon | Greece | 37.65 | 21.33333 |
| Mykonos | Greece | 37.45 | 25.33333 |
| Nauplia | Greece | 37.56667 | 22.8 |
| Patmos | Greece | 37.3 | 26.58333 |
| Piraeus | Greece | 37.93333 | 23.63333 |
| Preveza | Greece | 38.95 | 20.75 |
| Sifnos Is. | Greece | 36.96667 | 24.71667 |
| Skaramanga | Greece | 38 | 23.58333 |
| Syros | Greece | 37.43333 | 24.95 |
| Thessaloniki | Greece | 40.63333 | 22.93333 |
| Thira | Greece | 36.41667 | 25.45 |
| Zante | Greece | 37.78333 | 20.9 |
| Aasiaat | Greenland | 68.71667 | -52.88333 |
| Ammassalik | Greenland | 65.58333 | -37.5 |
| Nanortalik | Greenland | 60.13333 | -45.25 |
| Narsaq | Greenland | 60.9 | -45.98333 |
| Nuuk | Greenland | 64.16667 | -51.73333 |
| Sisimiut | Greenland | 66.95 | -53.68333 |
| Pointe a Pitre | Guadeloupe | 16.21667 | -61.53333 |
| Apra | Guam | 13.45 | 144.61667 |
| Puerto Quetzal | Guatemala | 13.93 | -90.79028 |
| Santo Tomas de Castilla | Guatemala | 15.69361 | -88.61528 |
| Conakry | Guinea | 9.5 | -13.71667 |
| Kamsar | Guinea | 10.65 | -14.61667 |
| Bissau | Guinea-Bissau | 11.85 | -15.58333 |
| Puerto Cortes | Honduras | 15.85 | -87.93333 |
| Akureyri | Iceland | 65.68333 | -18.05 |
| Neskaupstadur | Iceland | 65.15 | -13.68333 |
| Alang | India | 21.4 | 72.15 |
| Bedi | India | 22.51667 | 70.03333 |
| Belekeri | India | 14.7 | 74.25 |
| Beypore | India | 11.16667 | 75.8 |
| Chennai | India | 13.08333 | 80.28333 |
| Cuddalore | India | 11.7 | 79.76667 |
| Dahej | India | 21.71667 | 72.58333 |
| Dhamra | India | 20.78333 | 86.9 |
| Dighi | India | 18.25 | 72.91667 |
| Ennore | India | 13.23333 | 80.33333 |
| Haldia | India | 22.01667 | 88.08333 |
| Hazira | India | 21.1 | 72.65 |
| Jafarabad | India | 20.86667 | 71.36667 |
| Jakhau | India | 23.23333 | 68.58333 |
| Jamnagar Term. | India | 22.5 | 69.83333 |
| Jawaharlal Nehru | India | 18.93333 | 72.85 |
| Kakinada | India | 17 | 82.28333 |
| Kandla | India | 23.03333 | 70.21667 |
| Karwar | India | 14.81667 | 74.1 |
| Kolkata | India | 22.58333 | 88.35 |
| Krishnapatnam | India | 14.3 | 80.08333 |
| Magdalla | India | 21.13333 | 72.73333 |
| Mormugao | India | 15.41667 | 73.8 |
| Muldwarka | India | 20.75 | 70.65 |
| Mumbai | India | 18.9 | 72.81667 |
| Mundra | India | 22.9 | 69.7 |
| New Mangalore | India | 12.91667 | 74.8 |
| Panaji | India | 15.48333 | 73.81667 |
| Paradip | India | 20.26667 | 86.68333 |
| Pipavav | India | 20.98333 | 71.56667 |
| Porbandar | India | 21.6 | 69.6 |
| Port Okha | India | 22.46667 | 69.08333 |
| Revadanda | India | 18.55 | 72.9 |
| Sikka | India | 22.56667 | 69.8 |
| Tuticorin | India | 8.8 | 78.18333 |
| Visakhapatnam | India | 17.7 | 83.3 |
| Amamapare | Indonesia | -4.81667 | 136.96667 |
| Ambon | Indonesia | -3.7 | 128.16667 |
| Anyer | Indonesia | -6.03333 | 105.93333 |
| Balikpapan | Indonesia | -1.26667 | 116.81667 |
| Batu Ampar | Indonesia | 1.16667 | 104 |
| Belawan | Indonesia | 3.8 | 98.71667 |
| Bengkalis | Indonesia | 1.46667 | 102.1 |
| Bima | Indonesia | -8.41667 | 118.71667 |
| Bitung | Indonesia | 1.43333 | 125.18333 |
| Bojonegara | Indonesia | -5.95 | 106.08333 |
| Bontang | Indonesia | 0.1 | 117.48333 |
| Cigading | Indonesia | -6.01667 | 105.95 |
| Cirebon | Indonesia | -6.68333 | 108.55 |
| Dumai | Indonesia | 1.68333 | 101.45 |
| Gresik | Indonesia | -7.15 | 112.65 |
| Kabil | Indonesia | 1.08333 | 104.13333 |
| Kaimana | Indonesia | -3.65 | 133.73333 |
| Kalbut | Indonesia | -7.61667 | 113.98333 |
| Kota Baru | Indonesia | -3.23333 | 116.23333 |
| Kuala Tanjung | Indonesia | 3.35 | 99.48333 |
| Lembar | Indonesia | -8.56667 | 116.06667 |
| Lhokseumawe | Indonesia | 5.16667 | 97.15 |
| Makassar | Indonesia | -5.13333 | 119.4 |
| Merak | Indonesia | -5.93333 | 105.96667 |
| Muara Pantai | Indonesia | 1.98333 | 117.86667 |
| Palopo | Indonesia | -2.98333 | 120.21667 |
| Panjang | Indonesia | -5.46667 | 105.33333 |
| Pemangkat | Indonesia | 1.13333 | 108.91667 |
| Plaju | Indonesia | -2.98333 | 104.83333 |
| Pomalaa | Indonesia | -4.16667 | 121.58333 |
| Pontianak | Indonesia | -0.01667 | 109.35 |
| Sampit | Indonesia | -2.98333 | 113.05 |
| Satui | Indonesia | -3.81667 | 115.48333 |
| Sekupang | Indonesia | 1.13333 | 103.93333 |
| Sintete | Indonesia | 1.21667 | 109.05 |
| Sungei Pakning | Indonesia | 1.36667 | 102.18333 |
| Tahuna | Indonesia | 3.58333 | 125.5 |
| Tanjung Bara | Indonesia | 0.53333 | 117.63333 |
| Tanjung Uban | Indonesia | 1.06667 | 104.21667 |
| Tarahan | Indonesia | -5.66667 | 105.46667 |
| Tarakan | Indonesia | 3.28333 | 117.6 |
| Teluk Bayur | Indonesia | -1 | 100.35 |
| Tuban | Indonesia | -6.76667 | 111.95 |
| Uleelheue | Indonesia | 5.55 | 95.28333 |
| Asaluyeh Term. | Iran | 27.51667 | 52.53333 |
| Bandar Abbas | Iran | 27.13333 | 56.2 |
| Bandar Anzali | Iran | 37.43333 | 49.48333 |
| Bandar Imam Khomeini | Iran | 30.41667 | 49.06667 |
| Bandar Mahshahr | Iran | 30.46667 | 49.18333 |
| Bandar Shahid Rejaie | Iran | 27.1 | 56.06667 |
| Bushire | Iran | 28.98333 | 50.83333 |
| Chah Bahar | Iran | 25.33333 | 60.53333 |
| Jask | Iran | 25.65 | 57.76667 |
| Kharg Is. | Iran | 29.23333 | 50.31667 |
| Lavan Is. | Iran | 26.78333 | 53.33333 |
| Basrah | Iraq | 30.5 | 47.81667 |
| Khor al Zubair | Iraq | 30.18333 | 47.9 |
| Umm Qasr | Iraq | 30.03333 | 47.95 |
| Cork | Ireland | 51.9 | -8.46667 |
| Dublin | Ireland | 53.35 | -6.21667 |
| Fenit | Ireland | 52.26667 | -9.85 |
| Foynes | Ireland | 52.61667 | -9.1 |
| Greenore | Ireland | 54.03333 | -6.13333 |
| Killybegs | Ireland | 54.63333 | -8.43333 |
| Kilrush | Ireland | 52.63333 | -9.5 |
| Ashdod | Israel | 31.82583 | 34.65417 |
| Ashkelon | Israel | 31.66667 | 34.55 |
| Eilat | Israel | 29.55 | 34.95 |
| Hadera | Israel | 32.45 | 34.91667 |
| Haifa | Israel | 32.81667 | 35 |
| Ancona | Italy | 43.61722 | 13.51167 |
| Augusta | Italy | 37.2 | 15.21667 |
| Avola | Italy | 36.9 | 15.15 |
| Brindisi | Italy | 40.65 | 17.98333 |
| Cagliari | Italy | 39.2 | 9.08333 |
| Civitavecchia | Italy | 42.1 | 11.8 |
| Crotone | Italy | 39.08333 | 17.13333 |
| Fiumicino | Italy | 41.76667 | 12.23333 |
| Genoa | Italy | 44.41667 | 8.91667 |
| Gioia Tauro | Italy | 38.43333 | 15.9 |
| La Spezia | Italy | 44.11667 | 9.83333 |
| Marina di Carrara | Italy | 44.03333 | 10.05 |
| Milazzo | Italy | 38.21667 | 15.25 |
| Monfalcone | Italy | 45.78333 | 13.55 |
| Naples | Italy | 40.85 | 14.26667 |
| Olbia | Italy | 40.91667 | 9.56667 |
| Ortona | Italy | 42.35 | 14.41667 |
| Piombino | Italy | 42.93333 | 10.55 |
| Porto Torres | Italy | 40.83333 | 8.4 |
| Porto Vesme | Italy | 39.2 | 8.4 |
| Pozzallo | Italy | 36.71667 | 14.85 |
| Ravenna | Italy | 44.41667 | 12.45 |
| Salerno | Italy | 40.68333 | 14.76667 |
| Santa Panagia | Italy | 37.11667 | 15.26667 |
| Sarroch | Italy | 39.08333 | 9.03333 |
| Savona | Italy | 44.3 | 8.48333 |
| Siracusa | Italy | 37.05 | 15.3 |
| Taranto | Italy | 40.45 | 17.2 |
| Termini Imerese | Italy | 37.98333 | 13.7 |
| Termoli | Italy | 42 | 15 |
| Torre Annunziata | Italy | 40.75 | 14.45 |
| Trieste | Italy | 45.65 | 13.8 |
| Venice | Italy | 45.43333 | 12.33333 |
| Rocky Point | Jamaica | 17.81667 | -77.15 |
| Aioi | Japan | 34.76667 | 134.46667 |
| Akita | Japan | 39.75 | 140.06667 |
| Amagasaki | Japan | 34.68333 | 135.38333 |
| Chiba | Japan | 35.56667 | 140.05 |
| Etajima | Japan | 34.23333 | 132.46667 |
| Fukuyama | Japan | 34.43333 | 133.45 |
| Funabashi | Japan | 35.66667 | 139.96667 |
| Gamagori | Japan | 34.8 | 137.21667 |
| Hachinohe | Japan | 40.53333 | 141.53333 |
| Hakata | Japan | 33.61667 | 130.38333 |
| Hamada | Japan | 34.88333 | 132.03333 |
| Hannan | Japan | 34.46667 | 135.35 |
| Haramichi | Japan | 37.63333 | 140.96667 |
| Hibi | Japan | 34.45 | 133.93333 |
| Higashi-Harima | Japan | 34.71667 | 134.83333 |
| Hikari | Japan | 33.95 | 131.93333 |
| Himeji | Japan | 34.76667 | 134.63333 |
| Hirohata | Japan | 34.76667 | 134.61667 |
| Hiroshima | Japan | 34.33333 | 132.45 |
| Hitachi | Japan | 36.48333 | 140.63333 |
| Hitachinaka | Japan | 36.41667 | 140.41667 |
| Hososhima | Japan | 32.45 | 131.66667 |
| Imari | Japan | 33.26667 | 129.81667 |
| Ishigaki | Japan | 24.33333 | 124.16667 |
| Ishikariwan Shinko | Japan | 43.21667 | 141.3 |
| Ishinomaki | Japan | 38.41667 | 141.3 |
| Itozaki | Japan | 34.38333 | 133.1 |
| Iwakuni | Japan | 34.16667 | 132.25 |
| Kagoshima | Japan | 31.58333 | 130.56667 |
| Kainan | Japan | 34.15 | 135.18333 |
| Kakogawa | Japan | 34.7 | 134.83333 |
| Kamaishi | Japan | 39.26667 | 141.9 |
| Kanazawa | Japan | 36.61667 | 136.6 |
| Kanda | Japan | 33.8 | 131 |
| Kasaoka | Japan | 34.5 | 133.5 |
| Kashima | Japan | 35.93333 | 140.7 |
| Kawasaki | Japan | 35.5 | 139.75 |
| Kesennuma | Japan | 38.86667 | 141.6 |
| Kiire | Japan | 31.38333 | 130.55 |
| Kinuura | Japan | 34.86667 | 136.95 |
| Kisarazu | Japan | 35.36667 | 139.88333 |
| Kobe | Japan | 34.66667 | 135.21667 |
| Kokura | Japan | 33.9 | 130.9 |
| Kudamatsu | Japan | 34 | 131.85 |
| Kure | Japan | 34.23333 | 132.55 |
| Kushiro | Japan | 42.98333 | 144.36667 |
| Matsushima | Japan | 32.93333 | 129.6 |
| Matsuura | Japan | 33.35 | 129.73333 |
| Matsuyama | Japan | 33.85 | 132.7 |
| Mega | Japan | 34.76667 | 134.7 |
| Mishima-Kawanoe | Japan | 34.01667 | 133.56667 |
| Misumi | Japan | 32.6 | 130.46667 |
| Mitajiri | Japan | 34.01667 | 131.6 |
| Miyako | Japan | 39.63333 | 141.96667 |
| Mizushima | Japan | 34.5 | 133.75 |
| Moji | Japan | 33.95 | 130.96667 |
| Mukaishima | Japan | 34.38333 | 133.2 |
| Muroran | Japan | 42.35 | 140.95 |
| Nagoya | Japan | 35.05 | 136.85 |
| Naha | Japan | 26.21667 | 127.66667 |
| Namikata | Japan | 34.11667 | 132.96667 |
| Nanao | Japan | 37.1 | 137.03333 |
| Niigata-Higashi | Japan | 38 | 139.23333 |
| Niihama | Japan | 33.98333 | 133.28333 |
| Ofunato | Japan | 38.98333 | 141.75 |
| Ogishima | Japan | 35.5 | 139.76667 |
| Oita | Japan | 33.26667 | 131.66667 |
| Omaezaki | Japan | 34.6 | 138.23333 |
| Onahama | Japan | 36.93333 | 140.91667 |
| Onomichi | Japan | 34.4 | 133.18333 |
| Osaka | Japan | 34.65 | 135.4 |
| Saganoseki | Japan | 33.25 | 131.86667 |
| Saiki | Japan | 32.96667 | 131.91667 |
| Sakaiminato | Japan | 35.53333 | 133.25 |
| Sakata | Japan | 38.93333 | 139.81667 |
| Sendai | Japan | 38.26667 | 141.05 |
| Shibushi | Japan | 31.46667 | 131.11667 |
| Shikama | Japan | 34.78333 | 134.66667 |
| Shimizu | Japan | 35.01667 | 138.53333 |
| Shimonoseki | Japan | 33.93333 | 130.93333 |
| Shimotsu | Japan | 34.11667 | 135.13333 |
| Shingu | Japan | 33.7 | 136 |
| Susaki | Japan | 33.38333 | 133.3 |
| Tachibana | Japan | 33.88333 | 134.71667 |
| Tagonoura | Japan | 35.13333 | 138.7 |
| Takehara | Japan | 34.31667 | 132.91667 |
| Tobata | Japan | 33.91667 | 130.85 |
| Tokachi | Japan | 42.3 | 143.33333 |
| Tokuyama | Japan | 34 | 131.8 |
| Tokyo | Japan | 35.68333 | 139.73333 |
| Tomakomai | Japan | 42.63333 | 141.63333 |
| Toyama | Japan | 36.75 | 137.23333 |
| Toyohashi | Japan | 34.71667 | 137.31667 |
| Tsu | Japan | 34.68333 | 136.55 |
| Tsukumi | Japan | 33.08333 | 131.86667 |
| Ube | Japan | 33.93333 | 131.2 |
| Uno | Japan | 34.48333 | 133.95 |
| Wakayama | Japan | 34.2 | 135.13333 |
| Yatsushiro | Japan | 32.51667 | 130.53333 |
| Yawata | Japan | 33.86667 | 130.8 |
| Yawatahama | Japan | 33.45 | 132.4 |
| Yokkaichi | Japan | 34.95 | 136.65 |
| Yokohama | Japan | 35.45 | 139.66667 |
| Yokoshima | Japan | 32.85 | 130.53333 |
| Yokosuka | Japan | 35.28333 | 139.68333 |
| Yura | Japan | 33.95 | 135.1 |
| St. Helier | Jersey | 49.18333 | -2.11667 |
| Aqaba | Jordan | 29.51667 | 35.01667 |
| Lamu | Kenya | -2.3 | 40.91667 |
| Mombasa | Kenya | -4.06667 | 39.68333 |
| Betio | Kiribati | 1.35 | 172.91667 |
| Mina Abdulla | Kuwait | 29.01667 | 48.16667 |
| Mina al Ahmadi | Kuwait | 29.06667 | 48.15 |
| Mina Saud | Kuwait | 28.75 | 48.43333 |
| Shuaiba | Kuwait | 29.03333 | 48.16667 |
| Liepaja | Latvia | 56.51667 | 21.01667 |
| Riga | Latvia | 56.96667 | 24.1 |
| Ventspils | Latvia | 57.4 | 21.55 |
| Beirut | Lebanon | 33.9 | 35.51667 |
| Chekka | Lebanon | 34.33333 | 35.73333 |
| Zahrani Term. | Lebanon | 33.53333 | 35.31667 |
| Monrovia | Liberia | 6.33333 | -10.81667 |
| Khoms | Libya | 32.65 | 14.26667 |
| Tobruk | Libya | 32.08333 | 23.98333 |
| Klaipeda | Lithuania | 55.71667 | 21.13333 |
| Mahajanga | Madagascar | -15.71667 | 46.31667 |
| Toamasina | Madagascar | -18.15 | 49.41667 |
| Tolagnaro | Madagascar | -25.03333 | 47 |
| Toliary | Madagascar | -23.36667 | 43.66667 |
| Bintulu | Malaysia | 3.16667 | 113.03333 |
| Kemaman | Malaysia | 4.18333 | 103.5 |
| Kerteh Term. | Malaysia | 4.51667 | 103.55 |
| Kota Kinabalu | Malaysia | 6 | 116.06667 |
| Kuantan | Malaysia | 3.96667 | 103.43333 |
| Kuching | Malaysia | 1.56667 | 110.4 |
| Kunak | Malaysia | 4.68333 | 118.25 |
| Labuan | Malaysia | 5.28333 | 115.23333 |
| Lahad Datu | Malaysia | 5.03333 | 118.33333 |
| Limbang | Malaysia | 4.75 | 115 |
| Malacca | Malaysia | 2.18333 | 102.25 |
| Miri | Malaysia | 4.38333 | 113.98333 |
| Pasir Gudang | Malaysia | 1.43333 | 103.9 |
| Penang | Malaysia | 5.41667 | 100.33333 |
| Port Dickson | Malaysia | 2.51667 | 101.78333 |
| Port Klang | Malaysia | 3 | 101.4 |
| Sandakan | Malaysia | 5.83333 | 118.13333 |
| Semporna | Malaysia | 4.48333 | 118.61667 |
| Sipitang | Malaysia | 5.08333 | 115.55 |
| Sungai Udang | Malaysia | 2.25 | 102.13333 |
| Tanjong Manis | Malaysia | 2.15 | 111.35 |
| Tanjung Langsat | Malaysia | 1.46667 | 104.01667 |
| Tanjung Pelepas | Malaysia | 1.35 | 103.55 |
| Tawau | Malaysia | 4.25 | 117.88333 |
| Teluk Ewa | Malaysia | 6.43333 | 99.75 |
| Teluk Intan | Malaysia | 4.01667 | 101.01667 |
| Male | Maldives | 4.16667 | 73.5 |
| Marsaxlokk | Malta | 35.81667 | 14.56667 |
| Valletta | Malta | 35.9 | 14.51667 |
| Kwajalein | Marshall Is. | 8.71667 | 167.73333 |
| Majuro Atoll | Marshall Is. | 7.08333 | 171.13333 |
| Fort de France | Martinique | 14.6 | -61.06667 |
| Nouadhibou | Mauritania | 20.9 | -17.05 |
| Point Central | Mauritania | 20.81667 | -17.03333 |
| Port de L'Amitie | Mauritania | 18 | -16.03333 |
| Port Louis | Mauritius | -20.15 | 57.48333 |
| Acapulco | Mexico | 16.83333 | -99.91667 |
| Altamira | Mexico | 22.48333 | -97.88333 |
| Coatzacoalcos | Mexico | 18.15 | -94.41667 |
| Dos Bocas | Mexico | 18.41667 | -93.13333 |
| Lazaro Cardenas | Mexico | 17.91667 | -102.18333 |
| Tampico | Mexico | 22.21667 | -97.88333 |
| Topolobampo | Mexico | 25.58333 | -109.05 |
| Veracruz | Mexico | 19.2 | -96.13333 |
| Bar | Montenegro | 42.08333 | 19.08333 |
| Casablanca | Morocco | 33.61667 | -7.6 |
| Jorf Lasfar | Morocco | 33.11667 | -8.63333 |
| Mohammedia | Morocco | 33.71667 | -7.36667 |
| Nador | Morocco | 35.26667 | -2.91667 |
| Safi | Morocco | 32.3 | -9.25 |
| Tangier | Morocco | 35.78333 | -5.8 |
| Beira | Mozambique | -19.83333 | 34.83333 |
| Maputo | Mozambique | -25.98333 | 32.6 |
| Nacala | Mozambique | -14.53333 | 40.66667 |
| Pemba | Mozambique | -12.95 | 40.5 |
| Quelimane | Mozambique | -17.88333 | 36.88333 |
| Thilawa | Myanmar | 16.66667 | 96.25 |
| Yangon | Myanmar | 16.76667 | 96.16667 |
| Luderitz | Namibia | -26.63333 | 15.15 |
| Walvis Bay | Namibia | -22.95 | 14.5 |
| Amsterdam | Netherlands | 52.36667 | 4.9 |
| Dordrecht | Netherlands | 51.8 | 4.65 |
| Eemshaven | Netherlands | 53.45 | 6.83333 |
| Europoort | Netherlands | 51.95 | 4.08333 |
| Hansweert | Netherlands | 51.45 | 4 |
| Harlingen | Netherlands | 53.18333 | 5.41667 |
| Hook of Holland | Netherlands | 51.98333 | 4.11667 |
| Maassluis | Netherlands | 51.91667 | 4.25 |
| Moerdijk | Netherlands | 51.7 | 4.61667 |
| Rotterdam | Netherlands | 51.9 | 4.48333 |
| Scheveningen | Netherlands | 52.1 | 4.26667 |
| Schiedam | Netherlands | 51.9 | 4.4 |
| Terneuzen | Netherlands | 51.33333 | 3.81667 |
| Vlaardingen | Netherlands | 51.9 | 4.35 |
| Wielingen | Netherlands | 51.38333 | 3.38333 |
| Ymuiden | Netherlands | 52.45 | 4.58333 |
| Zaandam | Netherlands | 52.43333 | 4.83333 |
| Bullen Bay | Netherlands Antilles | 12.18333 | -69.01667 |
| Caracas Bay | Netherlands Antilles | 12.06667 | -68.86667 |
| Kralendijk | Netherlands Antilles | 12.15 | -68.28333 |
| St. Michiel's Bay | Netherlands Antilles | 12.15 | -69.01667 |
| Noumea | New Caledonia | -22.28333 | 166.43333 |
| Auckland | New Zealand | -36.85 | 174.8 |
| Bluff | New Zealand | -46.61667 | 168.36667 |
| Lyttelton | New Zealand | -43.61667 | 172.71667 |
| Napier | New Zealand | -39.48333 | 176.91667 |
| Tauranga | New Zealand | -37.65 | 176.16667 |
| Timaru | New Zealand | -44.38333 | 171.25 |
| Wellington | New Zealand | -41.28333 | 174.76667 |
| Corinto | Nicaragua | 12.46667 | -87.18333 |
| Apapa-Lagos | Nigeria | 6.43333 | 3.4 |
| Bonny | Nigeria | 4.43333 | 7.15 |
| Brass Term. | Nigeria | 4.31667 | 6.23333 |
| Calabar | Nigeria | 4.96667 | 8.31667 |
| Forcados Term. | Nigeria | 5.16667 | 5.18333 |
| Okrika | Nigeria | 4.71667 | 7.08333 |
| Onne | Nigeria | 4.71667 | 7 |
| Port Harcourt | Nigeria | 4.76667 | 7 |
| Sapele | Nigeria | 5.9 | 5.68333 |
| Warri | Nigeria | 5.51667 | 5.73333 |
| Nampo | North Korea | 38.73333 | 125.41667 |
| Songjin | North Korea | 40.66667 | 129.2 |
| Saipan Is. | Northern Mariana Is. | 15.2 | 145.71667 |
| Borg Hbr. | Norway | 59.2 | 10.95 |
| Drammen | Norway | 59.73333 | 10.23333 |
| Egersund | Norway | 58.45 | 6 |
| Floro | Norway | 61.6 | 5.03333 |
| Haavik | Norway | 59.31667 | 5.31667 |
| Horten | Norway | 59.41667 | 10.5 |
| Hoyanger | Norway | 61.21667 | 6.06667 |
| Kaarsto | Norway | 59.26667 | 5.53333 |
| Kirkenes | Norway | 69.73333 | 30.05 |
| Kristiansund | Norway | 63.11667 | 7.73333 |
| Mo i Rana | Norway | 66.31667 | 14.13333 |
| Mongstad | Norway | 60.81667 | 5.03333 |
| Moss | Norway | 59.43333 | 10.66667 |
| Narvik | Norway | 68.43333 | 17.43333 |
| Slagen | Norway | 59.31667 | 10.53333 |
| Sunndalsora | Norway | 62.68333 | 8.6 |
| Duqm | Oman | 19.68333 | 57.73333 |
| Khasab | Oman | 26.21667 | 56.25 |
| Mina al Fahal | Oman | 23.65 | 58.53333 |
| Port Sultan Qaboos | Oman | 23.63333 | 58.56667 |
| Salalah | Oman | 16.93333 | 54 |
| Sohar | Oman | 24.5 | 56.63333 |
| Gadani Beach | Pakistan | 25.1 | 66.71667 |
| Gwadar | Pakistan | 25.1 | 62.4 |
| Karachi | Pakistan | 24.83333 | 67 |
| Port Muhammad Bin Qasim | Pakistan | 24.76667 | 67.35 |
| Balboa | Panama | 8.95 | -79.56667 |
| Chiriqui Grande | Panama | 8.96667 | -82.1 |
| Coco Solo | Panama | 9.36667 | -79.88333 |
| Cristobal | Panama | 9.35 | -79.91667 |
| Taboguilla Is. | Panama | 8.81667 | -79.51667 |
| Port Moresby | Papua New Guinea | -9.43333 | 147.1 |
| Cabo Blanco | Peru | -4.26667 | -81.25 |
| Callao | Peru | -12.05 | -77.15 |
| Chancay | Peru | -11.6 | -77.28333 |
| Conchan | Peru | -12.25 | -76.93333 |
| General San Martin | Peru | -13.83333 | -76.26667 |
| Ilo | Peru | -17.63333 | -71.35 |
| La Pampilla | Peru | -11.93333 | -77.18333 |
| Matarani | Peru | -16.98333 | -72.11667 |
| Paita | Peru | -5.08333 | -81.11667 |
| Puerto Bayovar | Peru | -5.78333 | -81.05 |
| Salaverry | Peru | -8.23333 | -79 |
| Supe | Peru | -10.83333 | -77.73333 |
| Batangas | Philippines | 13.75 | 121.05 |
| Cebu | Philippines | 10.3 | 123.9 |
| Davao | Philippines | 7.06667 | 125.61667 |
| General Santos | Philippines | 6.1 | 125.16667 |
| Iloilo | Philippines | 10.7 | 122.56667 |
| Manila | Philippines | 14.58333 | 120.96667 |
| Mariveles | Philippines | 14.41667 | 120.5 |
| Masinloc | Philippines | 15.53333 | 119.95 |
| Pagbilao | Philippines | 13.85 | 121.75 |
| Sangi | Philippines | 10.4 | 123.63333 |
| Subic Bay | Philippines | 14.78333 | 120.23333 |
| Villanueva | Philippines | 8.58333 | 124.75 |
| Darlowo | Poland | 54.43333 | 16.38333 |
| Gdansk | Poland | 54.35 | 18.65 |
| Gdynia | Poland | 54.53333 | 18.55 |
| Police | Poland | 53.55 | 14.6 |
| Swinoujscie | Poland | 53.93333 | 14.28333 |
| Szczecin | Poland | 53.41667 | 14.55 |
| Aveiro | Portugal | 40.65 | -8.75 |
| Horta | Portugal | 38.53333 | -28.63333 |
| Leixoes | Portugal | 41.18333 | -8.71667 |
| Lisbon | Portugal | 38.73333 | -9.11667 |
| Praia da Vitoria | Portugal | 38.73333 | -27.05 |
| Setubal | Portugal | 38.5 | -8.91667 |
| Sines | Portugal | 37.95 | -8.86667 |
| Viana do Castelo | Portugal | 41.68333 | -8.83333 |
| Halul Island Term. | Qatar | 25.66667 | 52.41667 |
| Mesaieed | Qatar | 24.91667 | 51.58333 |
| Ras Laffan | Qatar | 25.93333 | 51.53333 |
| Pointe des Galets | Reunion | -20.91667 | 55.28333 |
| Constantza | Romania | 44.16667 | 28.65 |
| Azov | Russia | 47.1 | 39.43333 |
| Baltiysk | Russia | 54.65 | 19.9 |
| De Kastri | Russia | 51.46667 | 140.78333 |
| Kaliningrad | Russia | 54.71667 | 20.51667 |
| Kavkaz | Russia | 45.33333 | 36.65 |
| Korsakov | Russia | 46.66667 | 142.75 |
| Kronshtadt | Russia | 60 | 29.76667 |
| Makhachkala | Russia | 42.98333 | 47.5 |
| Moskalvo | Russia | 53.58333 | 142.5 |
| Nakhodka | Russia | 42.8 | 132.9 |
| Novorossiysk | Russia | 44.73333 | 37.78333 |
| Petropavlovsk-Kamchatskiy | Russia | 53.01667 | 158.63333 |
| Poronaysk | Russia | 49.21667 | 143.11667 |
| Primorsk | Russia | 60.36667 | 28.63333 |
| Rostov | Russia | 47.16667 | 39.7 |
| St. Petersburg | Russia | 59.93333 | 30.3 |
| Tuapse | Russia | 44.08333 | 39.06667 |
| Uglegorsk | Russia | 49.06667 | 142.01667 |
| Ust-Luga | Russia | 59.66667 | 28.3 |
| Vanino | Russia | 49.08333 | 140.26667 |
| Vladivostok | Russia | 43.11667 | 131.88333 |
| Vostochnyy | Russia | 42.75 | 133.06667 |
| Dammam | Saudi Arabia | 26.5 | 50.2 |
| Gizan | Saudi Arabia | 16.9 | 42.53333 |
| Jeddah | Saudi Arabia | 21.46667 | 39.16667 |
| Jubail | Saudi Arabia | 27.01667 | 49.65 |
| Qadimah | Saudi Arabia | 22.35 | 39.08333 |
| Rabigh | Saudi Arabia | 22.75 | 38.98333 |
| Ras al Khafji | Saudi Arabia | 28.41667 | 48.58333 |
| Ras Tanura | Saudi Arabia | 26.63333 | 50.16667 |
| Yanbu | Saudi Arabia | 24.1 | 38.05 |
| Dakar | Senegal | 14.68333 | -17.41667 |
| Port Victoria | Seychelles | -4.61667 | 55.45 |
| Freetown | Sierra Leone | 8.5 | -13.21667 |
| Kissy | Sierra Leone | 8.48333 | -13.18333 |
| Changi | Singapore | 1.38333 | 103.98333 |
| Jurong Port | Singapore | 1.3 | 103.71667 |
| Pulau Bukom | Singapore | 1.23333 | 103.76667 |
| Singapore | Singapore | 1.26667 | 103.83333 |
| Koper | Slovenia | 45.55472 | 13.74139 |
| Berbera | Somalia | 10.45 | 45.01667 |
| Busan | South Korea | 35.1 | 129.06667 |
| Daesan | South Korea | 37.01667 | 126.41667 |
| Donghae | South Korea | 37.51361 | 129.12028 |
| Gojeong | South Korea | 36.3 | 126.45 |
| Gunsan | South Korea | 36 | 126.71667 |
| Gwangyang | South Korea | 34.9 | 127.7 |
| Hadong | South Korea | 34.95 | 127.81667 |
| Incheon | South Korea | 37.46667 | 126.6 |
| Jeju | South Korea | 33.51667 | 126.55 |
| Jinhae | South Korea | 35.13333 | 128.65 |
| Masan | South Korea | 35.18333 | 128.6 |
| Mokpo | South Korea | 34.78333 | 126.38333 |
| Mukho | South Korea | 37.54917 | 129.10861 |
| Okpo | South Korea | 34.88333 | 128.71667 |
| Pohang | South Korea | 36.03333 | 129.43333 |
| Pyeongtaek | South Korea | 37 | 126.78333 |
| Samchonpo | South Korea | 34.91667 | 128.06667 |
| Taean | South Korea | 36.91667 | 126.23333 |
| Tongyong | South Korea | 34.83333 | 128.41667 |
| Ulsan | South Korea | 35.48333 | 129.4 |
| Yosu | South Korea | 34.75 | 127.78333 |
| Alcanar | Spain | 40.58333 | 0.55 |
| Alcudia | Spain | 39.83333 | 3.13333 |
| Algeciras | Spain | 36.11667 | -5.43333 |
| Alicante | Spain | 38.33333 | -0.48333 |
| Almeria | Spain | 36.83333 | -2.5 |
| Aviles | Spain | 43.58333 | -5.93333 |
| Barcelona | Spain | 41.35 | 2.16667 |
| Bilbao | Spain | 43.31056 | -3.02861 |
| Cadiz | Spain | 36.5 | -6.33333 |
| Caraminal | Spain | 42.6 | -8.93333 |
| Carboneras | Spain | 37 | -1.88333 |
| Castellon | Spain | 39.96667 | 0.01667 |
| Ceuta | Spain | 35.88333 | -5.31667 |
| Corunna | Spain | 43.38333 | -8.36667 |
| Ferrol | Spain | 43.46667 | -8.26667 |
| Garrucha | Spain | 37.16667 | -1.83333 |
| Gijon | Spain | 43.55 | -5.66667 |
| Huelva | Spain | 37.26667 | -6.91667 |
| Las Palmas | Spain | 28.13306 | -15.43194 |
| Malaga | Spain | 36.71778 | -4.425 |
| Marin | Spain | 42.4 | -8.7 |
| Motril | Spain | 36.71667 | -3.51667 |
| Sagunto | Spain | 39.65 | -0.21667 |
| San Ciprian | Spain | 43.7 | -7.45 |
| Santa Cruz de Tenerife | Spain | 28.48333 | -16.23333 |
| Santander | Spain | 43.46667 | -3.76667 |
| Seville | Spain | 37.36667 | -6 |
| Tarragona | Spain | 41.1 | 1.23333 |
| Valencia | Spain | 39.45 | -0.3 |
| Vigo | Spain | 42.23333 | -8.66667 |
| Colombo | Sri Lanka | 6.9 | 79.86667 |
| Galle | Sri Lanka | 6.01667 | 80.21667 |
| Trincomalee | Sri Lanka | 8.56667 | 81.21667 |
| St. Eustatius | St. Kitts & Nevis | 17.48333 | -62.98333 |
| Port Sudan | Sudan | 19.61667 | 37.23333 |
| Paramaribo | Suriname | 5.83333 | -55.16667 |
| Paranam | Suriname | 5.6 | -55.08333 |
| Brofjorden | Sweden | 58.33333 | 11.38333 |
| Gefle | Sweden | 60.66667 | 17.16667 |
| Gothenburg | Sweden | 57.7 | 11.95 |
| Helsingborg | Sweden | 56.05 | 12.68333 |
| Karlshamn | Sweden | 56.16667 | 14.86667 |
| Karlskrona | Sweden | 56.16667 | 15.6 |
| Malmo | Sweden | 55.61667 | 13 |
| Norrkoping | Sweden | 58.6 | 16.2 |
| Skelleftea | Sweden | 64.73333 | 20.95 |
| Stenungsund | Sweden | 58.08333 | 11.81667 |
| Wallhamn | Sweden | 58 | 11.7 |
| Tartous | Syria | 34.9 | 35.86667 |
| An Ping | Taiwan | 22.98333 | 120.13333 |
| Hoping | Taiwan | 24.3 | 121.75 |
| Hualien | Taiwan | 24 | 121.63333 |
| Kaohsiung | Taiwan | 22.61667 | 120.25 |
| Keelung | Taiwan | 25.15 | 121.73333 |
| Mai-Liao | Taiwan | 23.78333 | 120.16667 |
| Suao | Taiwan | 24.6 | 121.86667 |
| Taichung | Taiwan | 24.28333 | 120.5 |
| Taipei | Taiwan | 25.16667 | 121.35 |
| Dar es Salaam | Tanzania | -6.81667 | 39.31667 |
| Kilwa Kivinje | Tanzania | -8.75 | 39.41667 |
| Mtwara | Tanzania | -10.25 | 40.2 |
| Tanga | Tanzania | -5.06667 | 39.1 |
| Zanzibar | Tanzania | -6.16667 | 39.18333 |
| Bangkok | Thailand | 13.7 | 100.56667 |
| Ko Sichang | Thailand | 13.15 | 100.8 |
| Krabi | Thailand | 8.06667 | 98.86667 |
| Laem Chabang | Thailand | 13.08333 | 100.88333 |
| Map Ta Phut | Thailand | 12.65 | 101.15 |
| Prachuap | Thailand | 11.18333 | 99.6 |
| Sattahip | Thailand | 12.65 | 100.85 |
| Siam Seaport | Thailand | 13.13333 | 100.88333 |
| Songkhla | Thailand | 7.23333 | 100.58333 |
| Sriracha | Thailand | 13.11667 | 100.88333 |
| Nassau | The Bahamas | 25.07444 | -77.32194 |
| South Riding Point | The Bahamas | 26.62556 | -78.22861 |
| Banjul | The Gambia | 13.45 | -16.56667 |
| Kpeme | Togo | 6.2 | 1.51667 |
| Lome | Togo | 6.13333 | 1.28333 |
| Chaguaramas | Trinidad & Tobago | 10.66667 | -61.65 |
| Point Fortin | Trinidad & Tobago | 10.18333 | -61.68333 |
| Point Lisas | Trinidad & Tobago | 10.4 | -61.5 |
| Pointe a Pierre | Trinidad & Tobago | 10.31667 | -61.46667 |
| Port of Spain | Trinidad & Tobago | 10.65 | -61.51667 |
| Gabes | Tunisia | 33.95 | 10.06667 |
| La Goulette | Tunisia | 36.81667 | 10.3 |
| La Skhira | Tunisia | 34.23333 | 10.06667 |
| Menzel Bourguiba | Tunisia | 37.15 | 9.8 |
| Sfax | Tunisia | 34.73333 | 10.76667 |
| Sousse | Tunisia | 35.83333 | 10.65 |
| Aliaga | Turkey | 38.8 | 26.98333 |
| Ambarli | Turkey | 40.96667 | 28.7 |
| Bandirma | Turkey | 40.35 | 27.96667 |
| Bozcaada | Turkey | 39.83333 | 26.06667 |
| Canakkale | Turkey | 40.15 | 26.41667 |
| Derince | Turkey | 40.75 | 29.81667 |
| Diliskelesi | Turkey | 40.76667 | 29.53333 |
| Dortyol | Turkey | 36.83333 | 36.23333 |
| Eregli | Turkey | 41.3 | 31.45 |
| Fethiye | Turkey | 36.63333 | 29.1 |
| Gelibolu | Turkey | 40.41667 | 26.68333 |
| Gemlik | Turkey | 40.43333 | 29.15 |
| Hereke | Turkey | 40.7 | 29.61667 |
| Hopa | Turkey | 41.41667 | 41.4 |
| Icdas | Turkey | 40.45 | 27.13333 |
| Iskenderun | Turkey | 36.6 | 36.16667 |
| Istanbul | Turkey | 41 | 28.96667 |
| Izmir | Turkey | 38.43333 | 27.13333 |
| Izmit | Turkey | 40.76667 | 29.91667 |
| Kusadasi | Turkey | 37.86667 | 27.23333 |
| Marmaris | Turkey | 36.85 | 28.26667 |
| Mersin | Turkey | 36.8 | 34.63333 |
| Nemrut Bay | Turkey | 38.75 | 26.91667 |
| Samsun | Turkey | 41.3 | 36.33333 |
| Tasucu | Turkey | 36.23333 | 33.98333 |
| Tekirdag | Turkey | 40.95 | 27.5 |
| Toros Gubre | Turkey | 36.91667 | 35.98333 |
| Trabzon | Turkey | 41 | 39.75 |
| Tutunciftlik | Turkey | 40.75 | 29.78333 |
| Tuzla | Turkey | 40.80917 | 29.34806 |
| Yalova | Turkey | 40.66667 | 29.25 |
| Yarimca | Turkey | 40.73333 | 29.76667 |
| Turkmenbashi | Turkmenistan | 40.01667 | 53 |
| Dneprobugskiy | Ukraine | 46.75 | 31.91667 |
| Ilichevsk | Ukraine | 46.3 | 30.65 |
| Kerch | Ukraine | 45.35 | 36.46667 |
| Kherson | Ukraine | 46.61667 | 32.6 |
| Mariupol | Ukraine | 47.05 | 37.5 |
| Nikolayev | Ukraine | 46.96667 | 31.96667 |
| Ochakov | Ukraine | 46.6 | 31.55 |
| Odessa | Ukraine | 46.48333 | 30.75 |
| Oktyabrsk | Ukraine | 46.83333 | 31.95 |
| Sevastopol | Ukraine | 44.61667 | 33.36667 |
| Theodosia | Ukraine | 45.03333 | 35.4 |
| Yuzhnyy | Ukraine | 46.6 | 31.01667 |
| Abu Dhabi | United Arab Emirates | 24.48333 | 54.36667 |
| Ajman | United Arab Emirates | 25.41667 | 55.43333 |
| Arzanah Is. | United Arab Emirates | 24.78333 | 52.56667 |
| Das Is. | United Arab Emirates | 25.15 | 52.86667 |
| Dubai | United Arab Emirates | 25.26667 | 55.26667 |
| Fujairah | United Arab Emirates | 25.16667 | 56.36667 |
| Hamriyah | United Arab Emirates | 25.46667 | 55.48333 |
| Jebel Ali | United Arab Emirates | 24.99028 | 55.06083 |
| Kalba | United Arab Emirates | 25.08333 | 56.33333 |
| Khor Fakkan | United Arab Emirates | 25.35 | 56.36667 |
| Mina Saqr | United Arab Emirates | 25.98333 | 56.05 |
| Ruwais | United Arab Emirates | 24.15 | 52.71667 |
| Sharjah | United Arab Emirates | 25.36667 | 55.38333 |
| Zirku Is. | United Arab Emirates | 24.88333 | 53.06667 |
| Belfast | United Kingdom | 54.6 | -5.93333 |
| Blyth | United Kingdom | 55.11667 | -1.48333 |
| Bristol | United Kingdom | 51.45 | -2.63333 |
| Canvey Is. | United Kingdom | 51.51667 | 0.63333 |
| Dover | United Kingdom | 51.11667 | 1.33333 |
| Falmouth | United Kingdom | 50.16667 | -5.05 |
| Fawley | United Kingdom | 50.81667 | -1.33333 |
| Felixstowe | United Kingdom | 51.95 | 1.31667 |
| Finnart | United Kingdom | 56.11667 | -4.83333 |
| Glasgow | United Kingdom | 55.86667 | -4.28333 |
| Hamble | United Kingdom | 50.81667 | -1.3 |
| Hull | United Kingdom | 53.75 | -0.3 |
| Hunterston | United Kingdom | 55.75 | -4.88333 |
| Immingham | United Kingdom | 53.63333 | -0.18333 |
| Irvine | United Kingdom | 55.6 | -4.68333 |
| Isle of Grain | United Kingdom | 51.43333 | 0.7 |
| Liverpool | United Kingdom | 53.41667 | -3 |
| London | United Kingdom | 51.5 | -0.06667 |
| Milford Haven | United Kingdom | 51.71278 | -5.06167 |
| Mistley | United Kingdom | 51.95 | 1.08333 |
| Newport | United Kingdom | 51.56667 | -2.98333 |
| Plymouth | United Kingdom | 50.36667 | -4.18333 |
| Port Talbot | United Kingdom | 51.58333 | -3.71667 |
| Portsmouth | United Kingdom | 50.8 | -1.1 |
| Rosyth | United Kingdom | 56.01667 | -3.45 |
| Ryde | United Kingdom | 50.73333 | -1.15 |
| Sheerness | United Kingdom | 51.43333 | 0.73333 |
| Shoreham | United Kingdom | 50.83333 | -0.25 |
| Southampton | United Kingdom | 50.9 | -1.4 |
| Southend | United Kingdom | 51.53333 | 0.71667 |
| Tees | United Kingdom | 54.58333 | -1.16667 |
| Teignmouth | United Kingdom | 50.55 | -3.5 |
| Thamesport | United Kingdom | 51.43333 | 0.7 |
| Tilbury | United Kingdom | 51.45 | 0.33333 |
| Tyne | United Kingdom | 55 | -1.43333 |
| Astoria | United States | 46.18333 | -123.83333 |
| Baltimore | United States | 39.28333 | -76.58333 |
| Barbers Point | United States | 21.26667 | -158.08333 |
| Baton Rouge | United States | 30.46667 | -91.18333 |
| Bayport | United States | 29.61667 | -95.01667 |
| Beaumont | United States | 30.08333 | -94.1 |
| Benicia | United States | 38.03333 | -122.15 |
| Brownsville | United States | 25.95 | -97.4 |
| Brunswick | United States | 31.13333 | -81.48333 |
| Bucksport | United States | 44.56667 | -68.8 |
| Burns Hbr. | United States | 41.63333 | -87.18333 |
| Burnside | United States | 30.13333 | -90.91667 |
| Cameron | United States | 29.78333 | -93.31667 |
| Chalmette | United States | 29.93333 | -90 |
| Charleston | United States | 32.78333 | -79.93333 |
| Cherry Point | United States | 48.86667 | -122.75 |
| Cleveland | United States | 41.51667 | -81.71667 |
| Coos Bay | United States | 43.38333 | -124.2 |
| Corpus Christi | United States | 27.8 | -97.38333 |
| Davant | United States | 29.6 | -89.85 |
| Delaware City | United States | 39.56667 | -75.58333 |
| Detroit | United States | 42.33333 | -83.03333 |
| Dutch Hbr. | United States | 53.9 | -166.53333 |
| El Segundo | United States | 33.9 | -118.45 |
| Erie | United States | 42.16667 | -80.08333 |
| Fernandina | United States | 30.68333 | -81.46667 |
| Galveston | United States | 29.3 | -94.8 |
| Gary Hbr. | United States | 41.6 | -87.35 |
| Geismar | United States | 30.21667 | -91.01667 |
| Honolulu | United States | 21.3 | -157.86667 |
| Houston | United States | 29.75 | -95.33333 |
| Jacksonville | United States | 30.38333 | -81.63333 |
| Kalama | United States | 46.01667 | -122.83333 |
| Lake Charles | United States | 30.21667 | -93.25 |
| Long Beach | United States | 33.75 | -118.21667 |
| Longview | United States | 46.13333 | -122.93333 |
| Los Angeles | United States | 33.71667 | -118.26667 |
| Marcus Hook | United States | 39.81667 | -75.41667 |
| Miami | United States | 25.78333 | -80.18333 |
| Milwaukee | United States | 43.05 | -87.86667 |
| Mobile | United States | 30.68333 | -88.05 |
| Morehead City | United States | 34.71667 | -76.7 |
| New Haven | United States | 41.25 | -72.9 |
| New Orleans | United States | 29.96667 | -90.08333 |
| New York | United States | 40.7 | -74.01667 |
| Newport News | United States | 36.96667 | -76.41667 |
| Oakland | United States | 37.8 | -122.28333 |
| Orange | United States | 30.1 | -93.73333 |
| Palm Beach | United States | 26.76667 | -80.05 |
| Pascagoula | United States | 30.35 | -88.56667 |
| Paulsboro | United States | 39.83333 | -75.25 |
| Pennsauken | United States | 39.96667 | -75.03333 |
| Pensacola | United States | 30.41667 | -87.21667 |
| Philadelphia | United States | 39.9 | -75.13333 |
| Plaquemines | United States | 30.28333 | -91.23333 |
| Point Comfort | United States | 28.63333 | -96.56667 |
| Port Angeles | United States | 48.13333 | -123.41667 |
| Port Arthur | United States | 29.86667 | -93.91667 |
| Port Canaveral | United States | 28.41667 | -80.58333 |
| Port Everglades | United States | 26.1 | -80.11667 |
| Port Hueneme | United States | 34.1525 | -119.20361 |
| Port Lavaca | United States | 28.61667 | -96.63333 |
| Port Manatee | United States | 27.5 | -82.55 |
| Port Neches | United States | 29.98333 | -93.93333 |
| Port Wentworth | United States | 32.1 | -81.15 |
| Providence | United States | 41.8 | -71.38333 |
| Sabine | United States | 29.71667 | -93.86667 |
| Sacramento | United States | 38.53333 | -121.5 |
| San Diego | United States | 32.70528 | -117.13778 |
| San Francisco | United States | 37.8 | -122.41667 |
| Savannah | United States | 32.08333 | -81.08333 |
| Searsport | United States | 44.45 | -68.91667 |
| Seattle | United States | 47.63333 | -122.33333 |
| Smith's Bluff | United States | 30 | -93.98333 |
| South Louisiana | United States | 30.1 | -90.48333 |
| St. Bernard | United States | 29.91667 | -89.91667 |
| Stockton | United States | 37.95 | -121.31667 |
| Tacoma | United States | 47.25 | -122.41667 |
| Tampa | United States | 27.95 | -82.45 |
| Texas City | United States | 29.36667 | -94.88333 |
| Montevideo | Uruguay | -34.9 | -56.26667 |
| Nueva Palmira | Uruguay | -33.88333 | -58.41667 |
| Amuay Bay | Venezuela | 11.76667 | -70.25 |
| Coloncha | Venezuela | 9.2 | -71.75 |
| El Palito | Venezuela | 10.48333 | -68.11667 |
| El Tablazo | Venezuela | 10.75 | -71.53333 |
| Guanta | Venezuela | 10.25 | -64.6 |
| La Guaira | Venezuela | 10.6 | -66.93333 |
| Maracaibo | Venezuela | 10.65 | -71.6 |
| Paradero | Venezuela | 8.4 | -62.63333 |
| Puerto Cabello | Venezuela | 10.48333 | -68 |
| Puerto Jose | Venezuela | 10.1 | -64.85 |
| Puerto la Cruz | Venezuela | 10.21667 | -64.63333 |
| Puerto Ordaz | Venezuela | 8.35 | -62.71667 |
| Punta Cardon | Venezuela | 11.6 | -70.25 |
| Cai Lan | Vietnam | 20.96667 | 107.03333 |
| Cam Pha | Vietnam | 21.01667 | 107.36667 |
| Da Nang | Vietnam | 16.1 | 108.3 |
| Haiphong | Vietnam | 20.86667 | 106.66667 |
| Ho Chi Minh City | Vietnam | 10.76667 | 106.71667 |
| Hongay | Vietnam | 20.95 | 107.05 |
| Nghe Tinh | Vietnam | 18.65 | 105.7 |
| Phu My | Vietnam | 10.58333 | 107.01667 |
| Qui Nhon | Vietnam | 13.76667 | 109.23333 |
| Vung Ang | Vietnam | 18.91667 | 106.4 |
| Vung Tau | Vietnam | 10.35 | 107.06667 |
| Limetree Bay | Virgin Is. | 17.69833 | -64.75611 |
| Laayoune | Western Sahara | 27.08333 | -13.43333 |
| Aden | Yemen | 12.8 | 44.96667 |
| Hodeidah | Yemen | 14.78333 | 42.95 |
| Mukalla | Yemen | 14.53333 | 49.13333 |
| Saleef | Yemen | 15.3 | 42.66667 |
