# Supplementary material for: Prioritising surveillance for alien organisms transported as stowaways on ships travelling to South Africa
Source: PLoS One. 2017 Apr 5;12(4):e0173340. doi: 10.1371/journal.pone.0173340 (PMC5381868; doi:10.1371/journal.pone.0173340)
Supplement: S2 Table — (DOCX) [file pone.0173340.s012.docx]

S2 Table. Species on the watch list developed for South Africa by Faulkner et al. (2014), the environment in which they occur (marine or terrestrial), whether they are found in climate zones or marine ecoregions that are associated with the source ports of high risk shipping routes, and if so whether they have a history of shipping-facilitated introduction.

| Species | Env. | Source port of high risk route | Ship introductions |
| --- | --- | --- | --- |
| *Abelmoschus moschatus* | Terrestrial | Yes | No |
| *Acacia concinna* | Terrestrial | Yes | Data n/a |
| *Acacia confusa* | Terrestrial | Yes | No |
| *Acacia mangium* | Terrestrial | Yes | Data n/a |
| *Acanthocereus tetragonus* | Terrestrial | Yes | Data n/a |
| *Acer platanoides* | Terrestrial | Yes | No |
| *Acridotheres fuscus* | Terrestrial | Yes | No |
| *Adenanthera pavonina* | Terrestrial | Yes | No |
| *Aedes aegypti* | Terrestrial | Yes | Data n/a |
| *Aegilops triuncialis* | Terrestrial | Yes | No |
| *Agrostis capillaris* | Terrestrial | Yes | Data n/a |
| *Akebia quinata* | Terrestrial | Yes | No |
| *Alliaria petiolata* | Terrestrial | Yes | No |
| *Alternanthera philoxeroides* | Terrestrial | Yes | Yes |
| *Ampelopsis brevipedunculata* | Terrestrial | Yes | No |
| *Andropogon virginicus* | Terrestrial | Yes | Data n/a |
| *Angiopteris evecta* | Terrestrial | Yes | No |
| *Annona glabra* | Terrestrial | Yes | No |
| *Annona squamosa* | Terrestrial | Yes | Data n/a |
| *Anolis carolinensis* | Terrestrial | Yes | Yes |
| *Asparagus officinalis* | Terrestrial | Yes | Data n/a |
| *Austroeupatorium inulifolium* | Terrestrial | Yes | Data n/a |
| *Bambusa vulgaris* | Terrestrial | Yes | No |
| *Bellis perennis* | Terrestrial | Yes | Data n/a |
| *Boehmeria penduliflora* | Terrestrial | Yes | Data n/a |
| *Boiga irregularis* | Terrestrial | Yes | Yes |
| *Bos taurus* | Terrestrial | Yes | No |
| *Bothriochloa pertusa* | Terrestrial | Yes | Data n/a |
| *Bubo virginianus* | Terrestrial | Yes | Data n/a |
| *Butomus umbellatus* | Terrestrial | Yes | Yes |
| *Calluna vulgaris* | Terrestrial | Yes | Data n/a |
| *Camelina sativa* | Terrestrial | Yes | Data n/a |
| *Canis latrans* | Terrestrial | Yes | Data n/a |
| *Canis lupus* | Terrestrial | Yes | No |
| *Carpodacus mexicanus* | Terrestrial | Yes | No |
| *Cavia porcellus* | Terrestrial | Yes | Data n/a |
| *Cecropia peltata* | Terrestrial | Yes | No |
| *Cenchrus echinatus* | Terrestrial | Yes | No |
| *Centaurea diffusa* | Terrestrial | Yes | No |
| *Cercopithecus mona* | Terrestrial | Yes | No |
| *Cestrum nocturnum* | Terrestrial | Yes | No |
| *Chamaeleo jacksonii* | Terrestrial | Yes | No |
| *Channa argus* | Terrestrial | Yes | No |
| *Channa marulius* | Terrestrial | Yes | No |
| *Chrysobalanus icaco* | Terrestrial | Yes | Data n/a |
| *Cinchona pubescens* | Terrestrial | Yes | No |
| *Cinnamomum verum* | Terrestrial | Yes | Data n/a |
| *Clarias batrachus* | Terrestrial | Yes | No |
| *Clematis vitalba* | Terrestrial | Yes | No |
| *Clidemia hirta* | Terrestrial | Yes | No |
| *Coccinia grandis* | Terrestrial | Yes | No |
| *Colubrina asiatica* | Terrestrial | Yes | No |
| *Corbicula fluminea* | Terrestrial | Yes | Yes |
| *Coronilla varia* | Terrestrial | Yes | No |
| *Crocidura suaveolens* | Terrestrial | Yes | Data n/a |
| *Cryphonectria parasitica* | Terrestrial | Yes | No |
| *Cryptostegia madagascariensis* | Terrestrial | Yes | No |
| *Culex quinquefasciatus* | Terrestrial | Yes | Yes |
| *Cynara cardunculus* | Terrestrial | Yes | Data n/a |
| *Cynoglossum officinale* | Terrestrial | Yes | Data n/a |
| *Cyprinella lutrensis* | Terrestrial | Yes | No |
| *Cytisus striatus* | Terrestrial | Yes | Data n/a |
| *Dioscorea bulbifera* | Terrestrial | Yes | No |
| *Dreissena polymorpha* | Terrestrial | Yes | Yes |
| *Dysdera crocata* | Terrestrial | Yes | Data n/a |
| *Elaeagnus angustifolia* | Terrestrial | Yes | No |
| *Elaeagnus pungens* | Terrestrial | Yes | Data n/a |
| *Elaeagnus umbellata* | Terrestrial | Yes | No |
| *Elaeis guineensis* | Terrestrial | Yes | No |
| *Elephantopus mollis* | Terrestrial | Yes | Data n/a |
| *Elettaria cardamomum* | Terrestrial | Yes | Data n/a |
| *Epipremnum pinnatum* | Terrestrial | Yes | Data n/a |
| *Equus caballus* | Terrestrial | Yes | Data n/a |
| *Erinaceus europaeus* | Terrestrial | Yes | No |
| *Erythrocebus patas* | Terrestrial | Yes | No |
| *Esox lucius* | Terrestrial | Yes | Data n/a |
| *Euglandina rosea* | Terrestrial | Yes | Data n/a |
| *Euonymus fortunei* | Terrestrial | Yes | No |
| *Eupatorium cannabinum* | Terrestrial | Yes | No |
| *Ficus rubiginosa* | Terrestrial | Yes | No |
| *Flemingia strobilifera* | Terrestrial | Yes | Data n/a |
| *Frangula alnus* | Terrestrial | Yes | No |
| *Fraxinus floribunda* | Terrestrial | Yes | Data n/a |
| *Gambusia holbrooki* | Terrestrial | Yes | No |
| *Gastrophryne carolinensis* | Terrestrial | Yes | Data n/a |
| *Gymnocoronis spilanthoides* | Terrestrial | Yes | No |
| *Haematoxylum campechianum* | Terrestrial | Yes | Data n/a |
| *Heliotropium angiospermum* | Terrestrial | Yes | Data n/a |
| *Hieracium pilosella* | Terrestrial | Yes | No |
| *Hiptage benghalensis* | Terrestrial | Yes | No |
| *Hydrocharis morsus-ranae* | Terrestrial | Yes | No |
| *Hygrophila polysperma* | Terrestrial | Yes | No |
| *Hypophthalmichthys nobilis* | Terrestrial | Yes | No |
| *Iguana iguana* | Terrestrial | Yes | No |
| *Impatiens glandulifera* | Terrestrial | Yes | No |
| *Lama guanicoe* | Terrestrial | Yes | Data n/a |
| *Lates niloticus* | Terrestrial | Yes | No |
| *Leiothrix lutea* | Terrestrial | Yes | Data n/a |
| *Lepidium latifolium* | Terrestrial | Yes | No |
| *Lepus europaeus* | Terrestrial | Yes | No |
| *Ligustrum robustum* | Terrestrial | Yes | No |
| *Limnocharis flava* | Terrestrial | Yes | No |
| *Limnophila sessiliflora* | Terrestrial | Yes | No |
| *Lithobates catesbeianus* | Terrestrial | Yes | No |
| *Luzula campestris* | Terrestrial | Yes | Data n/a |
| *Lymantria dispar* | Terrestrial | Yes | No |
| *Lymantria monacha* | Terrestrial | Yes | Data n/a |
| *Macaca fascicularis* | Terrestrial | Yes | Yes |
| *Macaca mulatta* | Terrestrial | Yes | No |
| *Melastoma candidum* | Terrestrial | Yes | No |
| *Merremia tuberosa* | Terrestrial | Yes | No |
| *Miconia calvescens* | Terrestrial | Yes | No |
| *Microstegium vimineum* | Terrestrial | Yes | No |
| *Mikania micrantha* | Terrestrial | Yes | No |
| *Mimosa diplotricha* | Terrestrial | Yes | No |
| *Miscanthus sinensis* | Terrestrial | Yes | No |
| *Misgurnus anguillicaudatus* | Terrestrial | Yes | No |
| *Molothrus ater* | Terrestrial | Yes | No |
| *Molothrus bonariensis* | Terrestrial | Yes | No |
| *Monomorium floricola* | Terrestrial | Yes | Data n/a |
| *Monomorium pharaonis* | Terrestrial | Yes | Yes |
| *Monopterus albus* | Terrestrial | Yes | No |
| *Montia fontana* | Terrestrial | Yes | Data n/a |
| *Mustela erminea* | Terrestrial | Yes | No |
| *Mustela nivalis* | Terrestrial | Yes | No |
| *Myiopsitta monachus* | Terrestrial | Yes | No |
| *Myriophyllum heterophyllum* | Terrestrial | Yes | No |
| *Najas minor* | Terrestrial | Yes | Yes |
| *Nasua nasua* | Terrestrial | Yes | Data n/a |
| *Natrix maura* | Terrestrial | Yes | Data n/a |
| *Neovison vison* | Terrestrial | Yes | No |
| *Neyraudia reynaudiana* | Terrestrial | Yes | No |
| *Ocimum gratissimum* | Terrestrial | Yes | Data n/a |
| *Onopordum acanthium* | Terrestrial | Yes | No |
| *Ovis ammon* | Terrestrial | Yes | Data n/a |
| *Ovis aries* | Terrestrial | Yes | No |
| *Oxyura jamaicensis* | Terrestrial | Yes | No |
| *Paederia foetida* | Terrestrial | Yes | No |
| *Passiflora maliformis* | Terrestrial | Yes | Data n/a |
| *Peromyscus fraterculus* | Terrestrial | Yes | Data n/a |
| *Peromyscus maniculatus* | Terrestrial | Yes | Data n/a |
| *Petromyzon marinus* | Terrestrial | Yes | Yes |
| *Phalloceros caudimaculatus* | Terrestrial | Yes | No |
| *Phragmites australis* | Terrestrial | Yes | No |
| *Pinus caribaea* | Terrestrial | Yes | Data n/a |
| *Pinus nigra* | Terrestrial | Yes | Data n/a |
| *Piper aduncum* | Terrestrial | Yes | Yes |
| *Pitangus sulphuratus* | Terrestrial | Yes | No |
| *Pluchea indica* | Terrestrial | Yes | Data n/a |
| *Podarcis sicula* | Terrestrial | Yes | Data n/a |
| *Polygala paniculata* | Terrestrial | Yes | Data n/a |
| *Polygonum cuspidatum* | Terrestrial | Yes | No |
| *Pomacea canaliculata* | Terrestrial | Yes | No |
| *Pomacea insularum* | Terrestrial | Yes | No |
| *Potamogeton crispus* | Terrestrial | Yes | No |
| *Potamogeton perfoliatus* | Terrestrial | Yes | No |
| *Potamopyrgus antipodarum* | Terrestrial | Yes | Yes |
| *Prosopis juliflora* | Terrestrial | Yes | Data n/a |
| *Pterygoplichthys anisitsi* | Terrestrial | Yes | No |
| *Pterygoplichthys pardalis* | Terrestrial | Yes | No |
| *Pycnonotus cafer* | Terrestrial | Yes | Yes |
| *Pyrus calleryana* | Terrestrial | Yes | No |
| *Python molurus bivittatus* | Terrestrial | Yes | No |
| *Ranunculus ficaria* | Terrestrial | Yes | No |
| *Rattus exulans* | Terrestrial | Yes | No |
| *Rauvolfia vomitoria* | Terrestrial | Yes | No |
| *Rhamnus alaternus* | Terrestrial | Yes | No |
| *Rhamnus cathartica* | Terrestrial | Yes | No |
| *Rhinella marina* | Terrestrial | Yes | Yes |
| *Rhizophora mangle* | Terrestrial | Yes | No |
| *Rhododendron ponticum* | Terrestrial | Yes | No |
| *Rhodomyrtus tomentosa* | Terrestrial | Yes | No |
| *Rhus longipes* | Terrestrial | Yes | Data n/a |
| *Rosa bracteata* | Terrestrial | Yes | Data n/a |
| *Rubus alceifolius* | Terrestrial | Yes | Data n/a |
| *Rubus ellipticus* | Terrestrial | Yes | No |
| *Rubus moluccanus* | Terrestrial | Yes | Data n/a |
| *Ruellia brevifolia* | Terrestrial | Yes | Data n/a |
| *Sagittaria sagittifolia* | Terrestrial | Yes | Data n/a |
| *Salix cinerea* | Terrestrial | Yes | No |
| *Samanea saman* | Terrestrial | Yes | Data n/a |
| *Scardinius erythrophthalmus* | Terrestrial | Yes | No |
| *Schismus arabicus* | Terrestrial | Yes | Data n/a |
| *Scinax ruber* | Terrestrial | Yes | Data n/a |
| *Scinax x-signatus* | Terrestrial | Yes | Data n/a |
| *Sechium edule* | Terrestrial | Yes | Data n/a |
| *Senecio squalidus* | Terrestrial | Yes | No |
| *Senecio viscosus* | Terrestrial | Yes | Data n/a |
| *Spartina densiflora* | Terrestrial | Yes | Data n/a |
| *Spermacoce verticillata* | Terrestrial | Yes | No |
| *Spiraea japonica* | Terrestrial | Yes | No |
| *Stellaria alsine* | Terrestrial | Yes | Data n/a |
| *Streptopelia decaocto* | Terrestrial | Yes | No |
| *Tabebuia heterophylla* | Terrestrial | Yes | Data n/a |
| *Tapinoma melanocephalum* | Terrestrial | Yes | No |
| *Terminalia catappa* | Terrestrial | Yes | No |
| *Thaumetopoea pityocampa* | Terrestrial | Yes | No |
| *Thunbergia grandiflora* | Terrestrial | Yes | Data n/a |
| *Tilapia mariae* | Terrestrial | Yes | No |
| *Trachycarpus fortunei* | Terrestrial | Yes | No |
| *Tridentiger trigonocephalus* | Terrestrial | Yes | Yes |
| *Tussilago farfara* | Terrestrial | Yes | No |
| *Typha latifolia* | Terrestrial | Yes | No |
| *Urochloa mutica* | Terrestrial | Yes | Data n/a |
| *Utricularia gibba* | Terrestrial | Yes | Data n/a |
| *Vallisneria spiralis* | Terrestrial | Yes | No |
| *Varanus indicus* | Terrestrial | Yes | No |
| *Verbascum thapsus* | Terrestrial | Yes | No |
| *Vespa velutina nigrithorax* | Terrestrial | Yes | Data n/a |
| *Vitex rotundifolia* | Terrestrial | Yes | No |
| *Viverricula indica* | Terrestrial | Yes | Data n/a |
| *Vulpes vulpes* | Terrestrial | Yes | No |
| *Wisteria sinensis* | Terrestrial | Yes | No |
| *Zosterops japonicus* | Terrestrial | Yes | No |
| *Acanthogobius flavimanus* | Terrestrial | Yes | Yes |
| *Acer ginnala* | Terrestrial | Yes | No |
| *Adelges piceae* | Terrestrial | Yes | No |
| *Ameiurus nebulosus* | Terrestrial | Yes | No |
| *Anoplophora glabripennis* | Terrestrial | Yes | Yes |
| *Berberis buxifolia* | Terrestrial | Yes | No |
| *Berberis darwinii* | Terrestrial | Yes | No |
| *Branta canadensis* | Terrestrial | Yes | No |
| *Bythotrephes longimanus* | Terrestrial | Yes | Yes |
| *Caiman crocodilus* | Terrestrial | Yes | No |
| *Callithrix jacchus* | Terrestrial | Yes | Data n/a |
| *Castor canadensis* | Terrestrial | Yes | No |
| *Celastrus orbiculatus* | Terrestrial | Yes | No |
| *Centaurea biebersteinii* | Terrestrial | Yes | Yes |
| *Cichla ocellaris* | Terrestrial | Yes | No |
| *Circus approximans* | Terrestrial | Yes | Data n/a |
| *Compsilura concinnata* | Terrestrial | Yes | Data n/a |
| *Crassula helmsii* | Terrestrial | Yes | No |
| *Cryptococcus fagisuga* | Terrestrial | Yes | No |
| *Didymosphenia geminata* | Terrestrial | Yes | Yes |
| *Eriocheir sinensis* | Terrestrial | Yes | Yes |
| *Falcataria moluccana* | Terrestrial | Yes | No |
| *Fuchsia magellanica* | Terrestrial | Yes | Data n/a |
| *Gunnera manicata* | Terrestrial | Yes | No |
| *Gunnera tinctoria* | Terrestrial | Yes | No |
| *Gymnocephalus cernuus* | Terrestrial | Yes | Yes |
| *Gymnorhina tibicen* | Terrestrial | Yes | No |
| *Heracleum mantegazzianum* | Terrestrial | Yes | No |
| *Hieracium aurantiacum* | Terrestrial | Yes | No |
| *Hieracium floribundum* | Terrestrial | Yes | Data n/a |
| *Hyphantria cunea* | Terrestrial | Yes | No |
| *Ips typographus* | Terrestrial | Yes | No |
| *Kalanchoe pinnata* | Terrestrial | Yes | Data n/a |
| *Lachnellula willkommii* | Terrestrial | Yes | No |
| *Leuciscus idus* | Terrestrial | Yes | No |
| *Linyphia triangularis* | Terrestrial | Yes | No |
| *Litoria aurea* | Terrestrial | Yes | No |
| *Lumbricus rubellus* | Terrestrial | Yes | Yes |
| *Lumbricus terrestris* | Terrestrial | Yes | No |
| *Lupinus polyphyllus* | Terrestrial | Yes | Data n/a |
| *Maconellicoccus hirsutus* | Terrestrial | Yes | No |
| *Merremia peltata* | Terrestrial | Yes | No |
| *Morone americana* | Terrestrial | Yes | No |
| *Mustela furo* | Terrestrial | Yes | No |
| *Myrmica rubra* | Terrestrial | Yes | No |
| *Mytilopsis leucophaeata* | Terrestrial | Yes | Data n/a |
| *Neogobius melanostomus* | Terrestrial | Yes | Yes |
| *Nymphaea odorata* | Terrestrial | Yes | No |
| *Octolasion tyrtaeum* | Terrestrial | Yes | Data n/a |
| *Ophiostoma ulmi* | Terrestrial | Yes | No |
| *Orconectes virilis* | Terrestrial | Yes | No |
| *Pacifastacus leniusculus* | Terrestrial | Yes | No |
| *Phoxinus phoxinus* | Terrestrial | Yes | Data n/a |
| *Phyllostachys flexuosa* | Terrestrial | Yes | Data n/a |
| *Pittosporum tenuifolium* | Terrestrial | Yes | Data n/a |
| *Procyon lotor* | Terrestrial | Yes | Data n/a |
| *Pylodictis olivaris* | Terrestrial | Yes | No |
| *Rangia cuneata* | Terrestrial | Yes | Yes |
| *Rangifer tarandus* | Terrestrial | Yes | Data n/a |
| *Raoiella indica* | Terrestrial | Yes | No |
| *Rhithropanopeus harrisii* | Terrestrial | Yes | Yes |
| *Rupicapra rupicapra* | Terrestrial | Yes | Data n/a |
| *Rutilus rutilus* | Terrestrial | Yes | Data n/a |
| *Salix humboldtiana* | Terrestrial | Yes | Data n/a |
| *Salvelinus namaycush* | Terrestrial | Yes | No |
| *Salvinia minima* | Terrestrial | Yes | Yes |
| *Sansevieria trifasciata* | Terrestrial | Yes | Data n/a |
| *Scolytus multistriatus* | Terrestrial | Yes | Data n/a |
| *Spartina anglica* | Terrestrial | Yes | Yes |
| *Tenrec ecaudatus* | Terrestrial | Yes | Data n/a |
| *Tetropium fuscum* | Terrestrial | Yes | Data n/a |
| *Tibouchina urvilleana* | Terrestrial | Yes | Data n/a |
| *Tomicus piniperda* | Terrestrial | Yes | No |
| *Tradescantia spathacea* | Terrestrial | Yes | Data n/a |
| *Trichosurus vulpecula* | Terrestrial | Yes | Data n/a |
| *Vallisneria nana* | Terrestrial | Yes | Data n/a |
| *Vespula vulgaris* | Terrestrial | Yes | Yes |
| *Zizania latifolia* | Terrestrial | Yes | Yes |
| *Acromyrmex octospinosus* | Terrestrial | No |  |
| *Alosa pseudoharengus* | Terrestrial | No |  |
| *Anolis aeneus* | Terrestrial | No |  |
| *Anolis cristatellus* | Terrestrial | No |  |
| *Anolis distichus* | Terrestrial | No |  |
| *Anolis equestris* | Terrestrial | No |  |
| *Anolis garmani* | Terrestrial | No |  |
| *Anolis lineatus* | Terrestrial | No |  |
| *Anolis porcatus* | Terrestrial | No |  |
| *Anolis richardii* | Terrestrial | No |  |
| *Anolis trinitatis* | Terrestrial | No |  |
| *Anopheles quadrimaculatus* | Terrestrial | No |  |
| *Artemia franciscana* | Terrestrial | No |  |
| *Bactrocera tryoni* | Terrestrial | No |  |
| *Batillaria attramentaria* | Terrestrial | No |  |
| *Boa constrictor imperator* | Terrestrial | No |  |
| *Cardamine glacialis* | Terrestrial | No |  |
| *Castilla elastica* | Terrestrial | No |  |
| *Cecropia schreberiana* | Terrestrial | No |  |
| *Cenchrus polystachios* | Terrestrial | No |  |
| *Cichlasoma urophthalmus* | Terrestrial | No |  |
| *Cinara cupressi* | Terrestrial | No |  |
| *Citharexylum spinosum* | Terrestrial | No |  |
| *Clematis terniflora* | Terrestrial | No |  |
| *Ctenosaura similis* | Terrestrial | No |  |
| *Cupaniopsis anacardioides* | Terrestrial | No |  |
| *Cynanchum rossicum* | Terrestrial | No |  |
| *Dendroctonus valens* | Terrestrial | No |  |
| *Dioscorea oppositifolia* | Terrestrial | No |  |
| *Dreissena bugensis* | Terrestrial | No |  |
| *Eleutherodactylus coqui* | Terrestrial | No |  |
| *Eleutherodactylus johnstonei* | Terrestrial | No |  |
| *Eleutherodactylus planirostris* | Terrestrial | No |  |
| *Euonymus alata* | Terrestrial | No |  |
| *Fuchsia boliviana* | Terrestrial | No |  |
| *Gallus varius* | Terrestrial | No |  |
| *Glyptoperichthys gibbiceps* | Terrestrial | No |  |
| *Ischaemum polystachyum* | Terrestrial | No |  |
| *Lepus americanus* | Terrestrial | No |  |
| *Limnoperna fortunei* | Terrestrial | No |  |
| *Lonicera maackii* | Terrestrial | No |  |
| *Lycalopex griseus* | Terrestrial | No |  |
| *Martes melampus* | Terrestrial | No |  |
| *Morella faya* | Terrestrial | No |  |
| *Norops grahami* | Terrestrial | No |  |
| *Norops sagrei* | Terrestrial | No |  |
| *Nypa fruticans* | Terrestrial | No |  |
| *Odocoileus virginianus* | Terrestrial | No |  |
| *Opuntia cochenillifera* | Terrestrial | No |  |
| *Orconectes rusticus* | Terrestrial | No |  |
| *Oryctes rhinoceros* | Terrestrial | No |  |
| *Osteopilus septentrionalis* | Terrestrial | No |  |
| *Pachycondyla chinensis* | Terrestrial | No |  |
| *Persicaria perfoliata* | Terrestrial | No |  |
| *Petrogale inornata* | Terrestrial | No |  |
| *Phalanger orientalis* | Terrestrial | No |  |
| *Pimenta dioica* | Terrestrial | No |  |
| *Pluchea carolinensis* | Terrestrial | No |  |
| *Polistes chinensis antennalis* | Terrestrial | No |  |
| *Prunus campanulata* | Terrestrial | No |  |
| *Pseudodiaptomus inopinus* | Terrestrial | No |  |
| *Pterygoplichthys multiradiatus* | Terrestrial | No |  |
| *Puccinia psidii* | Terrestrial | No |  |
| *Senegalia catechu* | Terrestrial | No |  |
| *Solanum tampicense* | Terrestrial | No |  |
| *Solenopsis invicta* | Terrestrial | No |  |
| *Solenopsis richteri* | Terrestrial | No |  |
| *Sphaeroma quoianum* | Terrestrial | No |  |
| *Triphasia trifolia* | Terrestrial | No |  |
| *Vespula pensylvanica* | Terrestrial | No |  |
| *Wasmannia auropunctata* | Terrestrial | No |  |
| *Waterhousea floribunda* | Terrestrial | No |  |
| *Xiphophorus hellerii* | Terrestrial | No |  |
| *Asterias amurensis* | Marine | Yes | Yes |
| *Boonea bisuturalis* | Marine | Yes | No |
| *Carijoa riisei* | Marine | Yes | Yes |
| *Caulerpa taxifolia* | Marine | Yes | No |
| *Ceratostoma inornatum* | Marine | Yes | Data n/a |
| *Charybdis japonica* | Marine | Yes | Yes |
| *Crepidula fornicata* | Marine | Yes | Yes |
| *Gemma gemma* | Marine | Yes | No |
| *Geukensia demissa* | Marine | Yes | Yes |
| *Gymnodinium catenatum* | Marine | Yes | Yes |
| *Halophila stipulacea* | Marine | Yes | Yes |
| *Hemigrapsus sanguineus* | Marine | Yes | Yes |
| *Ilyanassa obsoleta* | Marine | Yes | No |
| *Littorina littorea* | Marine | Yes | Yes |
| *Musculista senhousia* | Marine | Yes | Yes |
| *Mya arenaria* | Marine | Yes | Yes |
| *Mytilopsis sallei* | Marine | Yes | Yes |
| *Potamocorbula amurensis* | Marine | Yes | Yes |
| *Pterois volitans* | Marine | Yes | Yes |
| *Rapana venosa* | Marine | Yes | Yes |
| *Sabella spallanzanii* | Marine | Yes | Yes |
| *Sargassum fluitans* | Marine | Yes | Yes |
| *Sargassum muticum* | Marine | Yes | No |
| *Schizoporella errata* | Marine | Yes | Yes |
| *Schizoporella unicornis* | Marine | Yes | No |
| *Sparus aurata* | Marine | Yes | No |
| *Styela clava* | Marine | Yes | Yes |
| *Tubastraea coccinea* | Marine | Yes | Yes |
| *Undaria pinnatifida* | Marine | Yes | Yes |
| *Urosalpinx cinerea* | Marine | Yes | Data n/a |
| *Zoobotryon verticillatum* | Marine | Yes | Data n/a |
| *Alitta succinea* | Marine | Yes | Yes |
| *Chthamalus proteus* | Marine | Yes | Yes |
| *Phyllorhiza punctata* | Marine | No |  |
| *Trididemnum solidum* | Marine | No |  |
