# Supplementary material for: Prioritising surveillance for alien organisms transported as stowaways on ships travelling to South Africa
Source: PLoS One. 2017 Apr 5;12(4):e0173340. doi: 10.1371/journal.pone.0173340 (PMC5381868; doi:10.1371/journal.pone.0173340)
Supplement: S3 Table — (DOCX) [file pone.0173340.s013.docx]

S3 Table. Marine and terrestrial watch list species that might be transported along the twenty shipping routes to each South African port with the highest relative contribution to marine and terrestrial establishment debt. In brackets is a description of each species.

| South African port | Marine species | Terrestrial species |
| --- | --- | --- |
| Richards Bay | *Alitta succinea* (annelid)  *Asterias amurensis* (sea star)  *Carijoa riisei* (coral)  *Charybdis japonica* (crustacean)  *Crepidula fornicata* (mollusc)  *Gymnodinium catenatum* (alga)  *Halophila stipulacea* (aquatic plant)  *Hemigrapsus sanguineus* (crustacean)  *Littorina littorea* (mollusc)  *Musculista senhousia* (mollusc)  *Mya arenaria* (mollusc)  *Mytilopsis sallei* (mollusc)  *Pterois volitans* (fish)  *Rapana venosa* (mollusc)  *Sabella spallanzanii* (annelid)  *Schizoporella errata* (bryozoan)  *Styela clava* (tunicate)  *Tubastraea coccinea* (coral) | *Alternanthera philoxeroides* (aquatic plant; herb)  *Boiga irregularis* (reptile)  *Butomus umbellatus* (aquatic plant)  *Corbicula fluminea* (mollusc)  *Culex quinquefasciatus* (insect)  *Macaca fascicularis* (mammal)  *Monomorium pharaonis* (insect)  *Najas minor* (aquatic plant)  *Pycnonotus cafer* (bird)  *Tridentiger trigonocephalus* (fish) |
| Durban | *Asterias amurensis* (sea star)  *Carijoa riisei* (coral)  *Charybdis japonica* (crustacean)  *Crepidula fornicata* (mollusc)  *Geukensia demissa* (mollusc)  *Gymnodinium catenatum* (alga)  *Halophila stipulacea* (aquatic plant)  *Hemigrapsus sanguineus* (crustacean)  *Littorina littorea* (mollusc)  *Musculista senhousia* (mollusc)  *Mya arenaria* (mollusc)  *Mytilopsis sallei* (mollusc)  *Potamocorbula amurensis* (mollusc)  *Pterois volitans* (fish)  *Rapana venosa* (mollusc)  *Sabella spallanzanii* (annelid)  *Sargassum fluitans* (alga)  *Schizoporella errata* (bryozoan)  *Styela clava* (tunicate)  *Tubastraea coccinea* (coral)  *Undaria pinnatifida* (aquatic plant/alga) | *Alternanthera philoxeroides* (aquatic plant; herb)  *Boiga irregularis* (reptile)  *Corbicula fluminea* (mollusc)  *Culex quinquefasciatus* (insect)  *Macaca fascicularis* (mammal)  *Monomorium pharaonis* (insect)  *Najas minor* (aquatic plant)  *Piper aduncum* (tree; shrub)  *Pycnonotus cafer* (bird)  *Rhinella marina* (amphibian)  *Tridentiger trigonocephalus* (fish) |
| Port Elizabeth | *Carijoa riisei* (coral)  *Charybdis japonica* (crustacean)  *Chthamalus proteus* (crustacean)  *Crepidula fornicata* (mollusc)  *Geukensia demissa* (mollusc)  *Gymnodinium catenatum* (alga)  *Halophila stipulacea* (aquatic plant)  *Hemigrapsus sanguineus* (crustacean)  *Littorina littorea* (mollusc)  *Musculista senhousia* (mollusc)  *Mya arenaria* (mollusc)  *Mytilopsis sallei* (mollusc)  *Pterois volitans* (fish)  *Rapana venosa* (mollusc)  *Sabella spallanzanii* (annelid)  *Sargassum fluitans* (alga)  *Schizoporella errata* (bryozoan)  *Styela clava* (tunicate)  *Tubastraea coccinea* (coral)  *Undaria pinnatifida* (aquatic plant/alga) | *Alternanthera philoxeroides* (aquatic plant; herb)  *Anolis carolinensis* (reptile)  *Anoplophora glabripennis* (insect)  *Butomus umbellatus* (aquatic plant)  *Bythotrephes longimanus* (crustacean)  *Centaurea biebersteinii* (herb)  *Corbicula fluminea* (mollusc)  *Culex quinquefasciatus* (insect)  *Didymosphenia geminata* (alga)  *Dreissena polymorpha* (mollusc)  *Eriocheir sinensis* (crustacean)  *Gymnocephalus cernuus* (fish)  *Lumbricus rubellus* (annelid)  *Macaca fascicularis* (mammal)  *Monomorium pharaonis* (insect)  *Najas minor* (aquatic plant)  *Neogobius melanostomus* (fish)  *Petromyzon marinus* (fish)  *Piper aduncum* (tree; shrub)  *Potamopyrgus antipodarum* (mollusc)  *Pycnonotus cafer* (bird)  *Rangia cuneata* (mollusc)  *Rhinella marina* (amphibian)  *Rhithropanopeus harrisii* (crustacean)  *Spartina anglica* (grass)  *Tridentiger trigonocephalus* (fish)  *Vespula vulgaris* (insect)  *Zizania latifolia* (grass) |
| Cape Town | *Asterias amurensis* (sea star)  *Carijoa riisei* (coral)  *Charybdis japonica* (crustacean)  *Crepidula fornicata* (mollusc)  *Geukensia demissa* (mollusc)  *Gymnodinium catenatum* (alga)  *Hemigrapsus sanguineus* (crustacean)  *Littorina littorea* (mollusc)  *Musculista senhousia* (mollusc)  *Mya arenaria* (mollusc)  *Mytilopsis sallei* (mollusc)  *Potamocorbula amurensis* (mollusc)  *Pterois volitans* (fish)  *Rapana venosa* (mollusc)  *Sabella spallanzanii* (annelid)  *Sargassum fluitans* (alga)  *Schizoporella errata* (bryozoan)  *Styela clava* (tunicate)  *Tubastraea coccinea* (coral)  *Undaria pinnatifida* (aquatic plant/alga) | *Acanthogobius flavimanus* (fish)  *Alternanthera philoxeroides* (aquatic plant; herb)  *Anolis carolinensis* (reptile)  *Anoplophora glabripennis* (insect)  *Boiga irregularis* (reptile)  *Butomus umbellatus* (aquatic plant)  *Bythotrephes longimanus* (crustacean)  *Centaurea biebersteinii* (herb)  *Corbicula fluminea* (mollusc)  *Culex quinquefasciatus* (insect)  *Dreissena polymorpha* (mollusc)  *Eriocheir sinensis* (crustacean)  *Gymnocephalus cernuus* (fish)  *Lumbricus rubellus* (annelid)  *Macaca fascicularis* (mammal)  *Monomorium pharaonis* (insect)  *Najas minor* (aquatic plant)  *Neogobius melanostomus* (fish)  *Petromyzon marinus* (fish)  *Piper aduncum* (tree; shrub)  *Potamopyrgus antipodarum* (mollusc)  *Pycnonotus cafer* (bird)  *Rangia cuneata* (mollusc)  *Rhinella marina* (amphibian)  *Rhithropanopeus harrisii* (crustacean)  *Spartina anglica* (grass)  *Tridentiger trigonocephalus* (fish)  *Vespula vulgaris* (insect) |
| Saldanha Bay | *Asterias amurensis* (sea star)  *Carijoa riisei* (coral)  *Charybdis japonica* (crustacean)  *Crepidula fornicata* (mollusc)  *Geukensia demissa* (mollusc)  *Gymnodinium catenatum* (alga)  *Halophila stipulacea* (aquatic plant)  *Hemigrapsus sanguineus* (crustacean)  *Littorina littorea* (mollusc)  *Musculista senhousia* (mollusc)  *Mya arenaria* (mollusc)  *Mytilopsis sallei* (mollusc)  *Potamocorbula amurensis* (mollusc)  *Pterois volitans* (fish)  *Rapana venosa* (mollusc)  *Sabella spallanzanii* (annelid)  *Sargassum fluitans* (alga)  *Schizoporella errata* (bryozoan)  *Styela clava* (tunicate)  *Tubastraea coccinea* (coral)  *Undaria pinnatifida* (aquatic plant/alga) | *Acanthogobius flavimanus* (fish)  *Alternanthera philoxeroides* (aquatic plant; herb)  *Boiga irregularis* (reptile)  *Corbicula fluminea* (mollusc)  *Culex quinquefasciatus* (insect)  *Macaca fascicularis* (mammal)  *Monomorium pharaonis* (insect)  *Najas minor* (aquatic plant)  *Piper aduncum* (tree; shrub)  *Pycnonotus cafer* (bird)  *Rhinella marina* (amphibian)  *Tridentiger trigonocephalus* (fish) |
